# The impact of charge on chlorpromazine interaction with lipid membranes

**DOI:** 10.1016/j.jlr.2026.101035

**Published:** 2026-04-06

**Authors:** Ana Gorse, Nicolò Paracini, Marion Mathelié-Guinlet, Victoria Lublin, Nada Taib-Maamar, Andrea Valentina Lopez Castillo, Yann Fichou, Estelle Rascol, Isabel D. Alves

**Affiliations:** 1 University Bordeaux, CNRS, Bordeaux INP, CBMN, UMR 5248, Pessac, France; 2 Institut Laue-Langevin, Large Scale Structures Group, 71 Avenue des Martyrs, Grenoble 38000, France; 3 Department of Pharmacy, Faculty of Health and Medical Sciences, University of Copenhagen, 2100 Copenhagen, Denmark

**Keywords:** drug therapy, brain lipids, schizophrenia, epidemiology, physical biochemistry, phospholipids/phosphatidylcholine, phospholipids/phosphatidylserine

## Abstract

Psychiatric disorders, such as schizophrenia, are treated with antipsychotics. One such molecule is chlorpromazine (CPZ) that mainly exerts its therapeutic effect via its antagonist activity on the dopamine D2 receptor. Due to its high lipophilicity, it can partition and accumulate in membranes, which can lead to a delayed full clinical effect, reached weeks after the beginning of treatment. CPZ’s insertion in membranes impacts the bilayer’s physicochemical properties, which can indirectly impact membrane-embedded receptors, such as the D2 receptor. To further delve into the potential role of phosphatidylserine (PS) in CPZ-membrane interaction and subsequent remodeling of membrane properties, we have investigated CPZ interactions with phosphatidylcholine bilayers, a common model zwitterionic lipid, in the absence and presence of 10% PS. The study provides a comprehensive overview of CPZ’s preferential interaction with PS-containing membranes. Altogether, data from complementary techniques showed a higher affinity of CPZ for PS headgroups, enhanced PS-mediated penetration in the membrane, and higher drug retention within the anionic lipid bilayer compared with POPC alone. At high concentration, CPZ induced the formation of mixed micelles on the surface of the treated membranes.

## Graphical abstract

**Figure:**
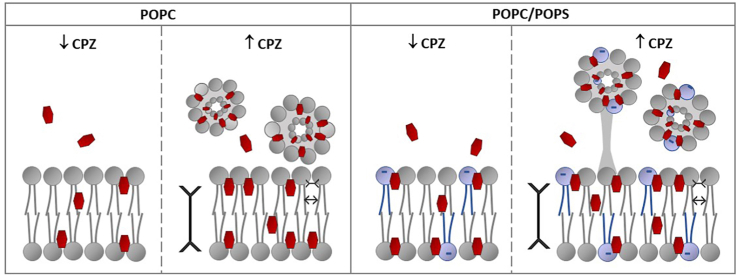

Chlorpromazine (CPZ) is a first-generation antipsychotic (AP) used in the treatment of psychotic illnesses, such as schizophrenia. The drug alleviates positive symptoms of schizophrenia caused by hyperactive dopaminergic signaling and mostly achieves its effect by blocking the D2 dopaminergic receptor (D2R) (1, 2, 3, 4) Its introduction to the market as the first AP in the 1950s has greatly improved the treatment of patients affected by psychiatric disorders (5, 6).

The drug’s affinity for lipid membranes arises from its strong lipophilic character, which promotes its preferential localization in a lipid environment rather than in the hydrophilic cytoplasm, as shown by the smaller percentage found in the latter (7). Besides the plasma membrane, where CPZ and other APs have been reported to accumulate, certain lipophilic APs interact with and accumulate in hydrophobic lipid compartments, including lipoprotein particles in blood cells as well as intracellularly in lipid droplets and related organelles (8, 9, 10, 11, 12, 13, 14). While the drug’s main clinical effect occurs via binding to D2R, CPZ accumulation in membranes may, in part, explain the drug’s delayed therapeutic onset and the persistence of its effects after discontinuing therapy (9). The lipid composition may therefore influence CPZ’s membrane insertion and modulate the drug’s impact on membrane properties, such as water penetration, membrane thickness, and other physicochemical properties.

The effect of CPZ on various membrane models has been explored in previous studies. CPZ was shown to increase the thickness of liquid-disordered phases while decreasing that of liquid-ordered phases in phosphatidylcholine (PC)/sphingomyelin/cholesterol bilayers (10). It also modified the surface potential and thermotropic behavior of lipid membranes by abolishing the pretransition and decreasing the main lipid phase transition temperature in dipalmitoylphosphatidylcholine/phosphatidylserine (PS) vesicles (11, 15). Additionally, the drug’s toxic effect was observed in mitochondrial membranes, where CPZ disrupted mitochondrial respiration and potassium equilibrium (16). Similarly, drug concentrations above 100 μM applied to rat brain slices induced pore formation, leading to destabilization of membrane integrity (17). These effects critically depend on *i*) the charge of CPZ, which is mostly protonated at physiological pH (p*K*a = 9.2) and *ii*) its capacity to aggregate (18). Indeed, while the molecule’s critical micelle concentration (the concentration above which micellization occurs) was reported to be between 0.1 and 0.2 mM, the presence of CPZ aggregates was seen at concentrations as low as 0.02 mM (19, 20).

In addition to altering membrane structure, CPZ impacts lipid metabolism, especially that of PS, a negatively charged phospholipid. Soon after the molecule’s commercialization, CPZ’s effect on D-amino acid oxidase enzyme inhibition was observed and more recently confirmed by Shishikura *et al*., resulting in a higher serine content in cell membranes (21, 22, 23, 24) The incubation of erythrocytes with CPZ increases PS exposure in the outer leaflet, a redistribution that could lead to higher red blood cell death rates, as certain macrophages contain receptors that recognize PS (7, 25). Consistent with these observations, CPZ demonstrates preferential affinity for PS, as probed by studies on Langmuir monolayers and by NMR, and increases the surface area of PS, indicating significant structural membrane rearrangements (11, 26, 27).

A more general observation from lipidomic studies revealed that AP-treated schizophrenic patients show an altered red blood cell lipid composition when compared with healthy subjects, with lower sphingomyelin and higher PS content (28). This is particularly relevant since the lipid environment also plays an important role in the pharmacology of G protein-coupled receptors (29). High-resolution structures and modeling studies demonstrated that, among other lipids, negative species reside in key locations at receptor/effector interfaces, thereby modulating subsequent signaling cascades (30, 31, 32). On the other hand, the presence of charge in the membrane can impact global membrane physicochemical properties, such as order parameter and phospholipid clustering, which in turn modulate membrane protein activity (33). In D2R lipid reconstitution assays, PC alone or in the presence of phosphatidylethanolamine failed to restore the original D2R ligand-binding properties, but PS addition recovered it (34). These findings highlight that CPZ-induced changes in PS content in psychiatric patients could impact CPZ/receptor binding properties and D2R pharmacology, thus influencing the drug efficiency and potential side effects.

The present study aims to further elucidate the impact of PS on CPZ-membrane interactions. To this end, we investigated two model membranes, pure PC and PC/PS mixed bilayers, by complementary experimental biophysical methods and molecular dynamics simulations. These combined approaches allowed us to assess how the presence of PS impacts CPZ membrane partitioning, its bilayer localization, and structural perturbations. Additionally, the data suggest that PS might modulate the formation of CPZ-induced micellar structures, which could impact CPZ bioavailability and membrane-associated effects of the drug.

## Materials and methods

### Materials

POPC, 1-palmitoyl-2-oleoyl-*sn*-glycero-3-phospho-L-serine (sodium salt) (POPS), 1-palmitoyl-d31-2-oleoyl-*sn*-glycero-3-phosphocholine (16:0-d31-18:1 PC), 1-palmitoyl-d31-2-oleoyl-*sn*-glycero-3-[phospho-L-serine] (sodium salt) (16:0-d31-18:1 PS), 1-palmitoyl-2-oleoyl-*sn*-glycero-3-phospho(tempo)choline (16:0-18:1 Tempo PC), 1-palmitoyl-2-stearoyl-(5-doxyl)-*sn*-glycero-3-phosphocholine (16:0-5 Doxyl PC), 1-palmitoyl-2-stearoyl-(10-doxyl)-*sn*-glycero-3-phosphocholine (16:0-10 Doxyl PC), and 1-palmitoyl-2-stearoyl-(16-doxyl)-*sn*-glycero-3-phosphocholine (16:0-16 Doxyl PC) were obtained dissolved in chloroform from Avanti Polar Lipids (Alabaster, AL). The fluorescent marker 6-dodecanoyl-*N*,*N*-dimethyl-2-naphthylamine (Laurdan fluorescent dye) and CPZ were obtained from Sigma-Aldrich.

### Lipid model system preparation

POPC and POPS were first dissolved in chloroform, and either pure POPC or a mixture of POPC/POPS (9/1 M ratio) was dried under nitrogen flow in order to form lipid films. They were consequently placed in a desiccator for at least 2 h to remove the traces of organic solvent. Lipid films were subsequently hydrated with the appropriate buffer (the composition is provided within each method), from which different types of vesicles were formed based on their use in different methods.

Multilamellar vesicles (MLVs) were formed by vigorously vortexing the liposome suspension for 2 min, followed by heating to 50–60 °C for 10 min (step repeated 2x). Small unilamellar vesicles (SUVs) were then obtained from MLVs by exposing the suspensions to a sonication step (probe-tip sonication, 3 cycles of 10 min, amplitude of 40%, and 3 s pulses).

Large unilamellar vesicles (LUVs) were formed by exposing the vesicles to 5 freeze-thaw cycles (liquid nitrogen –50 °C water bath) accompanied by vortexing between each cycle. The vesicles were then passed 11x through the membrane of a specific size (100 nm or 200 nm, depending on the method) by using an Avanti Polar Lipids extruder. The final lipid concentration was determined using a modified Fiske-Subbarow protocol by measuring inorganic phosphate concentrations (35).

### Atomic force microscopy

#### Bilayer preparation

SUVs were formed as described above using 20 mM Hepes and 100 mM NaCl, pH 7.0 (POPC), or 10 mM Hepes and 150 mM KCl, pH 7.0 (POPC/POPS), for lipid hydration. They were deposited (100 μl of 1 mg/ml) on freshly cleaved mica substrates for 1 h at 60 °C, which promoted a supported lipid bilayer (SLB) formation by the fusion and rupture of SUVs. The buffer used for vesicle formation was added if needed to prevent drying. Samples were then rinsed 5 to 10 times with a 10 mM Hepes and 150 mM KCl (pH 7.0) buffer to remove unadsorbed vesicles. For POPC/POPS bilayers, 1 mM of CaCl_2_ was added upon incubation to facilitate the SUV fusion and rupture onto the mica hydrophilic surface.

#### CPZ incubation

Once the formation and stability over time of SLBs have been confirmed by atomic force microscopy (AFM) imaging, SLBs were incubated with CPZ at various concentrations (10, 50, and 500 μM) for 1 h at room temperature, without rinsing. The effect of CPZ was thus assessed by comparing three regions of interest on the SLBs before and after exposure to the AP.

#### AFM experiments

AFM imaging was performed using the PeakForce Quantitative Nanomechanics mode on an Icon setup (Bruker). Silicon nitride lever-C probes with a nominal spring constant of 0.24 N/m were used and calibrated before each experiment with the thermal noise method. All experiments were conducted in 10 mM Hepes and 150 mM KCl (pH 7.0). AFM images (10 μm × 10 μm) were acquired with a scan rate of 0.7–1 Hz, a peakforce amplitude of 50–100 nm, and a peakforce frequency of 1 kHz, and the applied force was kept as low as possible to minimize damage (<1 nN). Force spectroscopy data were collected through force volume mapping, with 64 force curves (FCs) acquired per region of interest, allowing the quantification of morphological and mechanical properties of SLBs. During indentation, the force increases gradually as the tip compresses the bilayer until eventual membrane breakthrough, observed as a sudden drop in the FC, followed by a force rise upon substrate contact. AFM images were processed with the Nanoscope Analysis software (Bruker), and FCs were analyzed and processed with the JPKSPM Data Processing software (Bruker).

### Electron paramagnetic resonance

The PC-based spin labels were used to study changes in lipid membranes in CPZ presence. With a paramagnetic moiety providing the electron paramagnetic resonance (EPR) signal attached to either the polar headgroup (T-PC) or the alkyl chain carbon atoms (n-PC) of PC, they report information about the motion of the lipid molecules. T-PC, 5-PC, 10-PC, and 16-PC labels were used, providing information about the water-membrane interface and the inner portion of the lipid bilayer (36).

#### Liposome preparation

Lipids were premixed as described above along with a 1% molar ratio of a specific spin probe (either T-PC, 5-PC, 10-PC, or 16-PC), in the absence or presence of a 10% molar ratio of CPZ (dissolved in ethanol) that was premixed with lipids at this step. The mixtures were then dried as described above and subsequently hydrated in 20 μl buffer (25 mM Hepes, 150 mM KCl, pH 7.4) with the total lipid concentration of 30 mM and 3 mM CPZ in AP-containing samples. MLVs were formed as described above. Samples were placed in a 0.8 mm ID capillary for room-temperature measurements and in a 4 mm tube for cryogenic measurements.

Continuous-wave EPR was performed at X-band frequency (*ν* ≈ 9.75 GHz) on a Bruker ELEXSYS E500 using a cavity ER4102ST. Each sample was measured at room temperature and at cryotemperature 77 K using a liquid nitrogen bath cryostat filled with liquid nitrogen, under an argon atmosphere to prevent water condensation. The experimental parameters were microwave power 6.3 mW, modulation amplitude 0.1 mT, and conversion time 20 ms.

Hyperfine coupling A_Z_ was obtained from the spectra measured at cryotemperature by measuring the distance between the first and last EPR line and assigning it to twice the A_Z_ value, 2A_Z_ (Supplemental Fig. S12) (37). To obtain accurate values, a Gaussian was locally fitted to each peak. The uncertainty of the Gaussian position in the fit was reported as the error bar in the A_Z_ plots.

### Neutron reflectometry measurements

Neutron reflectometry (NR) exploits the large difference in scattering length density (SLD, proportional to the neutron refractive index) between hydrogen and deuterium to resolve the structure and composition of interfacial membrane parts having different SLD (38, 39). Selective deuteration of the lipid chains is therefore used to provide information about the impact of nondeuterated CPZ on membrane structure and drug penetration. Due to limitations in the availability of deuterium-labeled compounds, this deuteration scheme principally allows the resolution of CPZ penetration in the fatty acid region of the bilayer. Reflectivity measurements were performed on the time-of-flight FIGARO reflectometer at the European Neutron Source of the Institut Laue-Langevin (40).

#### Sample preparation

SLBs were prepared by vesicle fusion on atomically flat silicon crystals (50 × 80 × 15 m^3^) housed in solid-liquid cells. Silicon substrates were cleaned by two 30 min UV/ozone cycles, each followed by extensive rinsing with Milli-Q water, and assembled into solid-liquid measurement cells. SUVs composed of single-chain deuterated phospholipids (dPOPC or dPOPC/dPOPS 9/1) were prepared as described above. Lipid films were hydrated in Milli-Q water (1.5 mg/ml), and MLVs prepared were first sonicated in a bath sonicator for 10–20 min and then by using a tip sonicator as described above. The vesicles were then diluted to a final concentration of 0.1 mg/ml supplemented with a 2 mM CaCl_2_ solution before injection in the solid-liquid cell.

Reflectivity was measured under multiple isotopic buffer solutions, referred to as contrasts: 100% H_2_O, 100% D_2_O, silicon-matched water (38% D_2_O, 62% H_2_O), and four-matched water (66% D_2_O, 34% H_2_O), all containing 25 mM Hepes, 150 mM KCl, pH 7.4. These contrasts, corresponding to SLD values of −0.56 × 10^−6^Å^−2^(H_2_O), 2.07·× 10^−6^Å^−2^ (silicon-matched water), 4.00·× 10^−6^Å^−2^ (four-matched water), and 6.35·× 10^−6^Å^−2^ (D_2_O), were exchanged in the cell via a programmable HPLC pump and were then constrained to fit the same physical model of the interface during the data analysis (41). CPZ solutions at different concentrations (1, 5, 10, 25, 50, 100, 250, and 500 μM) were prepared in the H_2_O contrast for the titration experiments.

#### NR measurements

Liposome suspension (8 ml) was injected in the solid-liquid cell (internal volume ∼2 ml) to form the lipid bilayer that was measured in four solution contrasts. Contrasts containing CPZ were then injected at the concentration specified in the text and measured under the same conditions. The bilayers were also measured after rinsing off CPZ to investigate the reversibility of CPZ effects on the model membranes. Samples were measured at two incident angles (0.7° and 3.0°) with neutron wavelengths ranging from 2 Å to 20 Å at an instrument resolution $\frac{\Delta q}{q}$ of 7%, covering a total *q* range spanning from 0.008 Å^−1^ to 0.3 Å^−1^, where *q* is given by:

$$q=\frac{4\pi }{\lambda }*\sin\left(\theta \right)\left(\text{Equation}\;1\right)$$

Data reduction, including normalization to the direct beams, λ to *q* conversion, background subtraction, and stitching of the two angles into a single reflectivity curve, was performed using the LAMP software and the COSMOS interface.

#### Neutron reflectivity data analysis

NR curves were analyzed using the refnx software (42). POPC and POPC/POPS (9/1) bilayers were measured under multiple isotopic contrasts in three conditions: before CPZ addition (bilayer alone), in the presence of 500 μM CPZ (bilayer + CPZ, with CPZ present in all contrasts), and after rinsing off CPZ (rinsed bilayer). Parameter uncertainties were estimated using Markov Chain Monte Carlo (MCMC) sampling implemented in the refnx analysis package. Following the initial least-squares fit obtained through differential evolution optimization, MCMC sampling was performed to explore the posterior probability distribution of the fitted parameters. The sampling procedure consisted of an initial burn-in phase of 200 steps to allow the chains to reach equilibrium, followed by a production run of 200 steps with a thinning factor of 10 (retaining every 10th sample) to reduce autocorrelation between successive samples. The MCMC algorithm employed a Bayesian framework where the likelihood function was defined by the Chi-squared statistic of the global objective function encompassing all constrained solution contrast conditions, with uniform priors defined by the parameter bounds. The resulting posterior distributions, visualized through corner plots, provide both the most probable parameter values and their associated uncertainties, accounting for parameter correlations. Below, we describe the analysis strategy adopted to fit the NR data in the different conditions:

The pristine (blank) lipid bilayer was fitted to a slab model representing a symmetric bilayer modeled with five layers: *i*) native silicon oxide (SLD = 3.47 × 10^−6^Å^−2^), *ii*) inner phospholipid headgroup (SLD = 1.88 × 10^−6^Å^−2^), *iii*) inner phospholipid fatty acids (with the saturated chain deuterated, SLD = 3.17 × 10^−6^Å^−2^), *iv*) outer phospholipid fatty acids, and *v*) outer phospholipid headgroup, sandwiched between two semi-infinite layers representing the bulk silicon (SLD = 2.07 × 10^−6^Å^−2^) and the aqueous buffer solution (see SLD values listed above for each contrast condition). Each layer is defined by three primary parameters: thickness, SLD, and roughness, where the SLD of a chemical species is given by:

$$\sum_{i=1}^{n}\frac{b_{c}}{v_{m}}\left(\text{Equation}\;2\right)$$

with *b*_*c*_ being the coherent SLD of the ith atom within the molecular structure and *v*_*m*_ the molecular volume. A fourth hydration parameter modifies the SLD of each layer to account for the volume fraction of intercalated water in each layer depending on the solvent contrast, so that the sum of the volume fractions of each component in each layer is equal to 1. For the POPC bilayer, the best fit was obtained when an additional thin water layer was included between the silicon oxide and the inner headgroup region (Supplemental Fig. S3) whilst this was not needed to model the POPC/POPS bilayer (Supplemental Fig. S7). The presence of this water layer was also tested in the other models but was found to be consistently minimized to 0 Å thickness and was therefore not included in the analysis. The parameters relative to the silicon and silicon oxide layers obtained from the fit of the blank bilayers were assumed not to change in the following conditions and were therefore fixed to the values obtained from the blank bilayer fits.

To model the data measured on the bilayer + CPZ, a series of models were tested to find a physical representation of the interface that also fit the data satisfactorily (Supplemental Figs. S11, S4, S8). The models tested were, in order of complexity, *i*) the same pristine bilayer model without modifications to test whether effects could be modeled by a change in bilayer thickness or hydration, *ii*) the addition of a CPZ volume fraction intercalated in the fatty acid region of the bilayer, *iii*) the addition of tail-intercalated CPZ plus a uniform adsorbed layer above the bilayer, *iv*) the addition of tail-intercalated CPZ plus two adsorbed layers, and *v*) the addition of tail-intercalated CPZ plus three adsorbed layers. In these models, the thickness of the lipid headgroups and tails in each leaflet was kept symmetric, whereas the volume fractions of the components were allowed to vary independently between the inner and outer phospholipid monolayers. The process was repeated for the data collected on POPC and POPC/POPS. The models that gave the best results in terms of reduced χ^2^ were model (iii) for POPC (Fig. S5) and model (iv) for POPC/POPS (Supplemental Fig. S8). The results of the analysis with the discarded models are shown in Supplemental Fig. S11 together with the corresponding reduced χ^2^ values. Due to limited contrast between the SLD of the headgroup region (1.88·× 10^−6^Å^−2^) and the SLD of CPZ (1.30 · 10^−6^Å^−2^), we limited the models to fit CPZ penetration only in the fatty acid region of the model membranes during the initial model screening. After having identified the best models, we repeated the analysis by including CPZ in the headgroup region as well as in the tail regions in the selected models (Supplemental Figs. S5, S9).

After rinsing away CPZ, model (iii) was applied to both POPC and POPC/POPS model membranes since the adsorbed layer density was significantly reduced in both cases, and one adsorbed layer fits the data satisfactorily (Supplemental Figs. S6, S10). Given the significant reduction of CPZ left in the bilayer after rinsing and the lower information content that can be extracted from two contrasts compared with four, we only included CPZ in the tail region to fit the data after rinsing, which is where we are most sensitive to the presence of the drug.

All the values and associated errors obtained from the modeling are listed in Supplemental Tables S1–S4.

### Laurdan fluorescence spectroscopy

The Laurdan fluorescent probe is sensitive to the polarity of the environment and can therefore be used to follow polarity shifts in the bilayer in the presence of CPZ, mostly around the phospholipid glycerol group (43).

#### Lipid vesicle preparation

Lipids were premixed with a Laurdan probe dissolved in chloroform at a lipid/Laurdan molar ratio of 200/1. After lipid film formation, a 25 mM Hepes, 150 mM KCl, pH 7.4 buffer was used for hydration. LUVs of 200 nm were formed as described above, and the final lipid concentration was adjusted to 200 μM for the measurements. Two hundred microliters were put in separate wells using a black 96 flat-bottom well plate. After 1 h lipid incubation at 37 °C, different CPZ concentrations were added to separate wells (20 μl) with the final CPZ/lipid molar ratios of 1/5, 1/10, 1/25, 1/50, and 1/100. Twenty microliters of buffer were added for the control measurement. Thirty minutes after ligand addition, fluorescence emission intensity spectra were recorded in the 370–600 nm range using a 355 nm excitation wavelength. The measurements were done in triplicate.

Generalized polarization (GP) was then calculated using:

$$\text{GP}=\left(I_{440}-I_{490}\right)/\left(I_{440}+I_{490}\right)\left(\text{Equation}\;3\right)$$

where I_440_ represents the emission intensity at 440 nm (lipid-ordered phase) and I_490_ at 490 nm (disordered phase). This GP value reflects membrane order and hydration near the lipid headgroup region, as affected by dipolar relaxation and lipid environment (43).

### Intrinsic CPZ fluorescence spectroscopy

Partitioning of CPZ was evaluated using fluorescence spectroscopy, possible because of the presence of a phenothiazine ring in the CPZ structure, giving the AP fluorescence properties. The concentration of CPZ used was 40 μM, since its fluorescence emission is directly proportional to its concentration around this concentration (Supplemental Fig. S14).

#### Lipid vesicle preparation

Hepes (25 mM), 150 mM KCl, pH 7.4 buffer was used for the preparation of 100 nm LUVs, as described above.

#### Measurement

Different CPZ/lipid molar ratios were measured: 1/1, 1/2.5, 1/5, 1/7.5, 1/10, 1/15, and 1/25. Greiner black 96-well flat-bottom plates were used where 20 μl of CPZ was added to 180 μl of lipids—the final CPZ concentration was 40 μM. The fluorescence emission measurements were performed on a TECAN microplate reader with a 2 nm step between 390 and 600 nm, with the excitation at 320 nm. All spectra were background-subtracted (buffer alone), and the emission maximum at 420 nm was used to calculate the normalized intensity, I/I_w_ (I being the measured fluorescence intensity value and I_w_ the intensity of CPZ prior to lipid addition). I/I_w_ values were then plotted against total lipid concentration (0–1 mM), and the data obtained were consequently fitted using the simple partition model previously described to extract partition coefficients (K_P_) (44). This partition model assumes that CPZ is found either in the aqueous or in the lipid phase and is based on the following equation:

$$\frac{I}{I_{W}}=\frac{1+K_{P}*\gamma_{L}*\left[L\right]*I_{L}/I_{W}}{1+K_{P}*\gamma_{L}*\left[L\right]}\left(\text{Equation}\;4\right)$$

where γ_L_ represents the phospholipids’ molar volume (the value used is the value previously used for POPC: 0.763 M^−1^), [L] represents the total lipid concentration available for the interaction with CPZ, and I_L_ value displays the fluorescence intensity of CPZ if it was present 100% in the lipid phase (determined along with K_P_) (45, 46).

### All-atom molecular dynamics simulations

All simulations were performed using GROMACS (versions 2021.3 and 5.0.4) with the CHARMM36 force field and the TIP3P water model (47, 48, 49, 50, 51) CPZ parameters were generated via CGenFF. Despite moderate penalty scores, their reliability was confirmed by free energy perturbation calculations of octanol/water partition free energies, which matched well with DrugBank experimental data. The parameters were thus used without further modification.

A protonated CPZ was simulated in two membrane environments: *i*) a pure POPC bilayer and *ii*) a mixed bilayer with 90% POPC and 10% POPS. Each system included one CPZ molecule initially placed in the aqueous phase at ∼4.2 nm from the bilayer center.

Membranes were built using CHARMM-GUI with water slabs ≥2.25 nm on both sides. The POPC system (100 lipids) was solvated with 4,492 water molecules (box size: 5.84 × 5.84 × 8.50 nm^3^) and the mixed membrane (90 POPC + 10 POPS) with 7,492 water molecules (box: 5.81 × 5.81 × 11.32 nm^3^). Both systems were neutralized and ionized to 150 mM KCl (52).

Energy minimization was carried out using the steepest descent and conjugate gradient algorithms until the maximum force dropped below 100 kJ mol^-1^nm^-1^. Subsequently, five equilibration phases of 2 ns each were performed with decreasing position restraints applied to lipid and CPZ atoms (force constants: 1,000, 800, 400, 200, and 40 kJ mol^-1^nm^-2^). These steps were performed under constant temperature (300 K) and pressure (1 bar) using a Berendsen thermostat and semi-isotropic Berendsen barostat (53).

Production simulations were then carried out for 500 ns (two replicates per system) in the NPT ensemble, using a 2-fs time step. Temperature and pressure were maintained using the V-rescale thermostat and the Parrinello-Rahman barostat at 300 K and 1 bar, respectively, with a compressibility of 4.5 × 10^-5^bar^-1^ (54). Nonbonded interactions were handled using the Verlet cutoff scheme; long-range electrostatics were computed using the particle mesh Ewald method, and short-range interactions were truncated at 12 Å. Periodic boundary conditions were applied in all directions, and covalent bonds involving hydrogen atoms were constrained using the LINCS algorithm (55, 56, 57).

Density profiles were computed using the gmx density utility in GROMACS. Profiles were calculated along the bilayer normal (z) and centered with respect to the lipid molecules (POPC in the pure system; POPC + POPS in the mixed system) by applying the -center option. This procedure aligns each frame’s histogram relative to the instantaneous center of the bilayer, thereby correcting for drift and avoiding artificial profile broadening under semi-isotropic pressure coupling (58). The robustness of this approach for lipid bilayers has been demonstrated in recent computational studies (59, 60). Number density profiles (-dens number) were calculated with a bin width of 0.01 nm over the last 100 ns of the trajectories, with frames extracted every 10 ps. Profiles were obtained using the center of mass and normalized along the bilayer normal to account for variations in bilayer thickness and to allow direct comparison between systems.

To investigate the structural organization and preferential interactions between molecular species in our system, radial distribution function (RDF) analysis was performed using GROMACS. RDF, denoted as g(*r*), describes how the atomic density varies as a function of distance from a reference particle (in our case, CPZ molecule’s mass center), providing insight into short- and long-range ordering. The RDF was calculated using the gmx rdf tool, which computes the average number density of particle type B around a reference particle A (CPZ) as a function of radial distance *r*. The simulation trajectory was centered and corrected for periodic boundary conditions prior to analysis. Appropriate index groups were defined to target specific atom or molecule pairs of interest (P and N atoms and glycerol groups of POPC and POPS phospholipids). This analysis gives insight into molecular packing, hydration shells, and interaction preferences in complex biological and soft matter systems.

### Statistics

Statistical analyses were performed using GraphPad Prism (Laurdan GP values)/Origin software (AFM mechanical stability and rupture depth). One-way ANOVA (Dunnett’s multiple comparisons test) was used to compare Laurdan GP values between lipid alone and in the CPZ presence, and for AFM experiments, a two-sample Student’s *t*-test was used to compare the bilayers before and after CPZ incubation. *P* values between 0.05 and 0.01 are considered significant and presented with one star (∗), values between 0.01 and 0.001 are displayed with two stars (∗∗), and the ones smaller than 0.001 with three stars (∗∗∗).

## Results

### Negative lipids promote the restructuring of membranes following CPZ incubation

#### Atomic force microscopy

To investigate how CPZ impacts the morphological and mechanical behavior of lipid membranes, AFM experiments, both in imaging and force spectroscopy modes, were performed on POPC and POPC/POPS (9/1 M ratio) SLBs. Both bilayers initially exhibited a smooth and homogeneous surface that was not altered after exposure to a 1 h treatment under any of the tested CPZ concentrations (Supplemental Fig. S1A). However, AFM force spectroscopy revealed distinct changes in the mechanical behavior of the membranes in a CPZ concentration-dependent manner.

**Fig. 1:**
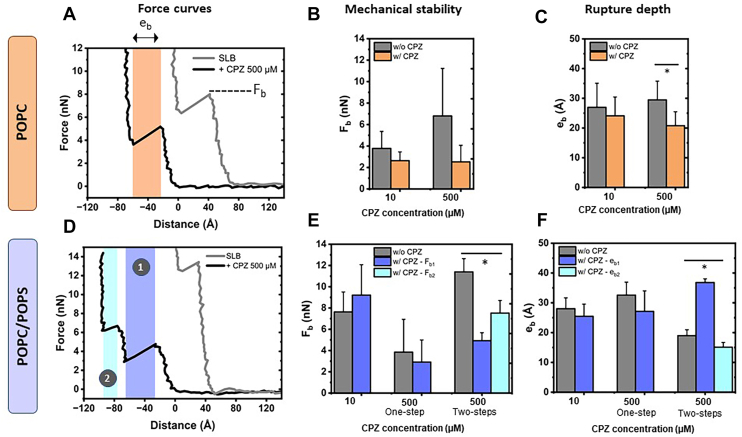
CPZ effect on membrane properties determined by AFM force spectroscopy. FC representation of POPC (A) and POPC/POPS (D) bilayers alone (gray) and after 500 μM CPZ incubation (black). A single breakthrough event seen for POPC with CPZ (A, orange) and a two-step breakthrough event observed for POPC/POPS are shown (D, first in dark blue and second in light blue). Mechanical stability (F_b_, B and E) and rupture depth (e_b_, C and F) are shown for separate bilayers (POPC top row, POPC/POPS bottom row) before (gray) and after incubation with 10 or 500 μM CPZ for POPC (orange) and POPC/POPS bilayer (dark blue, single or first breakthrough event and light blue, second breakthrough event just next to the mica substrate).

For pure POPC bilayers, FCs before and after CPZ treatment consistently exhibited the expected single breakthrough event, a typical signature indicating membrane rupture (Fig. 1A) (61). From these signatures, key parameters can be derived: *i*) the force at which the membrane ruptures and reflects its mechanical stability, named breakthrough force (F_b_) and *ii*) the vertical distance from the membrane surface to the substrate, reflecting membrane thickness after compression, named rupture distance (e_b_). At low concentrations of CPZ (10 μM), those membrane properties were not significantly modified in pure POPC bilayers (Fig. 1B). However, exposure to 500 μM of CPZ led to a slight, yet nonsignificant, decrease in the breakthrough force and a significant decrease of almost 10 Å in the rupture distance (Fig. 1C). This suggests that at high concentrations, CPZ induces membrane thinning, and eventual softening, without drastically altering the structural integrity of POPC membranes.

When anionic lipids were introduced in the SLBs, the classical “*one-step*” scenario described above was still observed after incubation with low concentrations of CPZ (10 μM) (Fig. 1A). At such a dose, both the mechanical stability and thickness of the membrane were not altered (Fig. 1E, F). Upon increasing CPZ concentration to 500 μM, the “*one-step*” scenario was only occasionally observed, revealing no significant changes in membrane properties induced by CPZ (Fig. 1E, F, “bars in the middle”). In contrast, another “*two-step*” scenario was predominant, where an initial rupture occurred at low force and large indentation depth, followed by a second event at higher force and lower indentation depth, characterized by distinct breakthrough forces (F_b1_< F_b2_) and rupture distances (e_b1_ >e_b2_) (Fig. 1E, F, “bars on the right”). In this specific scenario, 500 μM CPZ exposure led to a significant decrease in breakthrough forces (F_b1_ and F_b2_) and a significant increase in the rupture distance e_b1_. Such behavior suggests the formation of a two-layered membrane structure, with a less stable, thinner inner layer and a thicker outer layer. Interestingly, at intermediate concentration (50 μM), the “*two-step*” scenario was rarely observed (Supplemental Fig. S1D, E; only the “*two-step*” scenario is displayed), indicating that this CPZ-induced membrane restructuration critically depends on concentration. Furthermore, this effect was never observed in pure POPC bilayers, demonstrating that the presence of negatively charged POPS affects CPZ behavior and interactions at the membrane interface.

#### Neutron reflectometry

While AFM provides mechanical insight into drug-membrane interactions, it cannot probe, as a surface-sensitive technique, CPZ molecular localization and the structural changes it causes within the bilayer. To address this, specular NR provided key information on the distribution of bilayer components, inner and outer headgroup and fatty acid regions, CPZ, and water distribution profiles along the membrane normal.

**Fig. 2:**
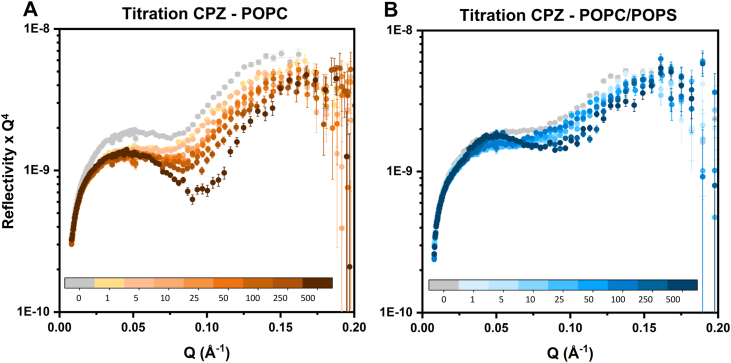
NR measurements of POPC (A) and POPC/POPS bilayers (B) as a function of CPZ concentration (titration). The *x*-axis represents the momentum transfer Q, and the *y*-axis represents the reflectivity values x Q^4^. The reflectivity from the pristine bilayer is plotted in gray, and the reflectivity curves in the presence of increasing CPZ concentrations injected on the bilayer are 1, 5, 10, 25, 50, 100, 250, and 500 μM, displayed from lighter to darker color, respectively. Measurements were performed in a buffer using 100% H_2_O as a solvent and containing the reported amount of dissolved CPZ. The error bars represent the standard deviation in the intensity measured at each q-point, following Poisson counting statistics.

Reflectivity was initially measured in a single contrast (H_2_O) on both POPC and POPC/POPS (9/1) bilayers at increasing CPZ concentrations, from 1 to 500 μM (Fig. 2). Interestingly, changes in the reflectivity, and thereby in the membrane’s scattering properties, were detected even at the lowest concentration of 1 μM (Fig. 2, *light orange/light blue* vs. *gray*). This indicates an early onset of interaction and structural changes because of CPZ, which occur in a concentration-dependent manner and are different for zwitterionic and anionic membranes. In the case of POPC (Fig. 2A), increasing CPZ concentration decreased the overall reflectivity intensity and shifted the minima to higher momentum transfer (Q) values, suggesting membrane thinning. In contrast, for POPC/POPS (Fig. 2B), reflectivity did not decrease monotonically, indicating more complex structural changes beyond simple variations in bilayer thickness and SLDs.

To further elucidate the structural effects of CPZ, the lipid bilayers were measured in four solution contrasts in the absence and presence of 500 μM CPZ, the concentration at which maximal effects on reflectivity were observed. Fitting all four contrasts simultaneously enables the determination of the models’ parameters and estimation of the values’ confidence, providing information on the membrane architecture at the interface. A simple slab model, reflecting symmetric bilayers (see Materials and methods section), confirmed >90% coverage for both POPC and POPC/POPS with expected headgroup and tail thickness between 7–9 Å and 17–18 Å, respectively, and a 45%–55% headgroup hydration (see Supplemental Table S1 for the complete list of parameters).

**Fig. 3:**
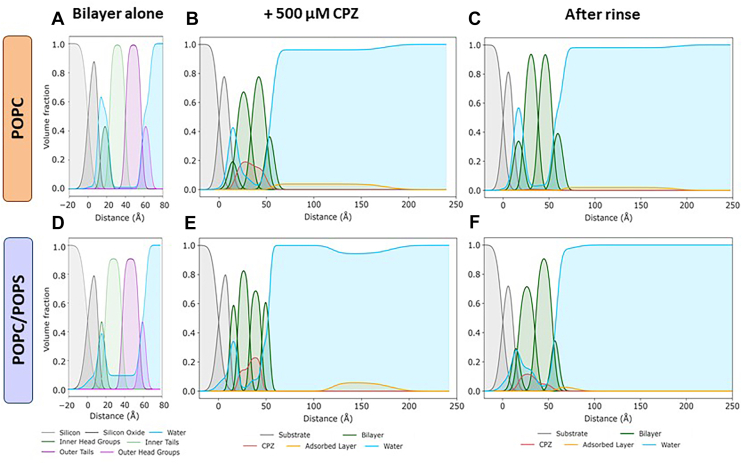
Volume fraction profiles of POPC (top) and POPC/POPS bilayer (bottom) for the bilayer alone (A and D, respectively), in the presence of 500 μM CPZ (B and E, respectively) and after rinse (C and F, respectively), obtained from NR measurements. The legend of the individual bilayer components is presented for the bilayer before CPZ addition, whereas after the addition of CPZ, all the bilayer components are all shown in green for clarity. Overall, NR revealed that CPZ in high concentration induces membrane thinning, and the formation of sparse aggregates above the bilayer. The interaction is stronger in the presence of negative lipids, which, unlike purely zwitterionic membranes, retain a considerable fraction of drug in the hydrophobic region of the bilayer upon rinsing. Those measurements thus complement AFM observations, further highlighting the structural rearrangement of bilayers induced by CPZ and its insertion, particularly at high concentration and in the presence of anionic lipids. Yet, as we only employed single-chain deuterated phospholipids, the hydrogen/deuterium contrast was reduced, especially in the highly hydrated headgroup region.

The simple bilayer model failed to properly fit the data of bilayers incubated with 500 μM CPZ, requiring models of increasing complexity (see Materials and methods and Supplemental data). The best-fit model revealed three main structural changes in both model membranes: *i*) bilayer thinning, *ii*) CPZ intercalation in the fatty acid region, and *iii*) formation of a sparse diffuse layer above the bilayer. For POPC, the total bilayer thickness decreased by ∼7 Å (from ∼53 Å to ∼46 Å), whereas for POPC/POPS, it decreased by ∼9 Å (from ∼51 Å to ∼42 Å), mainly via the fatty acid region. Penetration of CPZ in the hydrophobic region occurred in both bilayers to similar extents, yielding a final composition where occupancy of the drug amounted to 15%–25% of the dry volume of the fatty acid region of each lipid leaflet, with a higher total amount of CPZ accumulated in the POPS-containing membrane (Fig. 3B and E, Supplemental Table S2 and Supplemental Figs. S4, S8). As an additional control, we compared the reflectivity after 1 h and 3 h incubation with CPZ, which showed that the interaction was stable over time and that there was no time evolution of the interfacial structure in the time scale of the NR measurements after the addition of the drug (Supplemental Fig. S2).

Remarkably, NR analysis revealed a sparse layer of adsorbed material above both membranes with a low-volume fraction (∼4% for POPC and ∼6% for POPC/POPS) and with an SLD of ∼2.3 × 10^−6^Å^−2^, indicating the presence of a fraction of deuterated lipids in these structures, as the deuterated tails are the only component in the system to have an SLD larger than 2 × 10^−6^Å^−2^. In the POPC bilayer, the adsorbed layer was ∼119 Å thick and evenly distributed (Fig. 3B), whereas in POPC/POPS, it was found to be ∼61 Å but separated from the membrane by a solvent region of ∼66 Å (Fig. 3E).

In addition, the datasets collected in the presence of CPZ were tested against a model that allowed the presence of CPZ in the headgroup region to test whether information on CPZ penetration within this region could be extracted despite the low contrast between the drug and the lipid headgroups. For both membranes, allowing CPZ in the headgroup region did not improve the goodness of fit, and the obtained parameters’ probabilities showed broad distributions as expected from the lack of contrast (Supplemental Figs. S5 and S9, Supplemental Table S2) (62). However, whilst for POPC, the volume fractions were found to be skewed toward zero, in the case of POPC/POPS, the obtained values showed better-defined bell-shaped distributions. These differences are hints that CPZ might localize in the headgroup region of POPC/POPS to a higher extent than that of POPC membranes, although the data are not conclusive.

After rinsing, the diffuse and sparse adsorbed structures were significantly reduced in the POPC bilayer and largely removed from the POPC/POPS sample. In the latter, the rounded shape of the volume fraction profile (Fig. 3E) and the loose nature of the interaction suggest that the structure could correspond to rounded micelle-like structures of ∼61 Å in diameter, loosely connected to the bilayer. Rinsing removed all CPZ from the POPC bilayer, while the POPC/POPS membrane retained ∼45% of the initial CPZ (∼17% vs. initial ∼38%), strongly indicating higher drug retention by the anionic lipids (Supplemental Figs. S6, S10).

### Negative lipids impact membrane insertion, ordering, and polarity

#### Membrane partitioning by fluorescence spectroscopy

To understand the impact of charge on CPZ’s membrane partitioning, its intrinsic fluorescence, because of the cyclic π-conjugated ring, was used to determine its K_P_ between bulk solution and membrane environment. Adding 10% of negative lipid in the liposomes increased the partitioning of the molecule by approximately 70%, from 0.75 ± 0.14 × 10^4^ with the POPC-only model to 1.28 ± 0.22 × 10^4^ in the POPS presence, consistent with previous values obtained using identical lipid compositions and technique, isothermal titration calorimetry (1.2 × 10^4^ and 1.4 × 10^4^, respectively) (63). The partitioning curves are displayed in Supplemental Fig. S15.

#### Laurdan fluorescence spectroscopy

While NR reveals structural rearrangement at the membrane core level, CPZ’s impact on lipid packing and polarity at the headgroup region remains unclear. To address this, we used Laurdan fluorescence spectroscopy, which uses a solvatochromic probe reporting on the overall polarity and hydration at the glycerol backbone level of phospholipids. The general polarization (GP) parameter quantifies environment-dependent spectral shift (Equation 3; experimental section), thus providing a bulk estimate of lipid packing and local order (43, 64).

The data collected show a CPZ concentration-dependent increase in GP, indicating enhanced membrane ordering and dehydration of the headgroup region (Supplemental Fig. S13), which is in agreement with the reduced water content within the outer headgroup region observed by NR (Supplemental Tables S2–S3) (65). No significant difference between the two lipid compositions was detected.

#### Electron paramagnetic resonance

Since Laurdan provides an averaged response and cannot resolve depth-dependent changes, we employed EPR spectroscopy to gain site-specific insight into membrane dynamics. POPC phospholipids containing a spin probe located either on the headgroup or at different positions along the fatty acyl chains were used. This allowed us to assess how CPZ impacts specific regions of lipid molecules, depending on its proximity to the probes, providing indirect information on the depth of CPZ insertion into the bilayer.

**Fig. 4:**
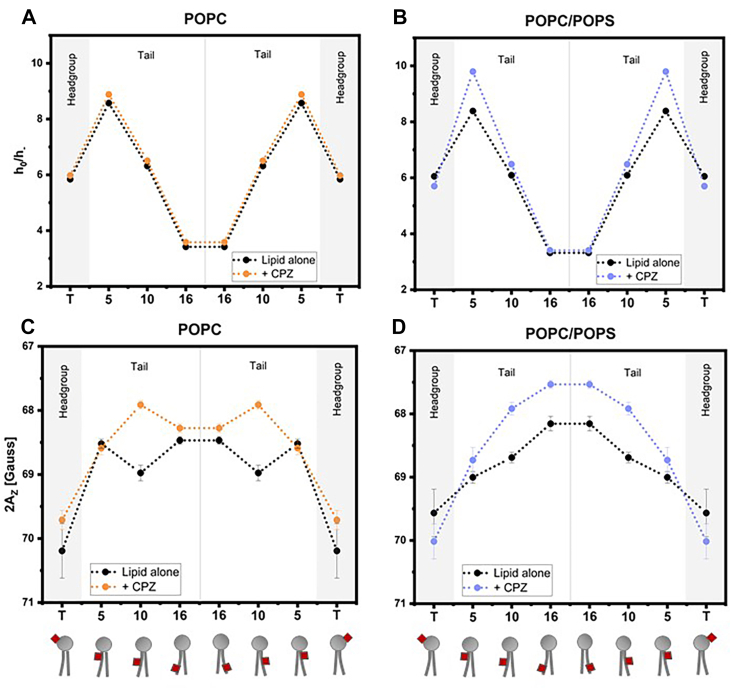
EPR spectral parameters obtained from spin probes placed along the lipid chains: TEMPO-PC, 5-PC, 10-PC, and 16-PC (shown as T, 5, 10, and 16, respectively) for POPC and POPS/POPS in the absence and presence of CPZ. The upper panels present the ratio of mid-field over high-field spectral line amplitudes (h_0_/h_−_), used as a reporter of label mobility, measured at room temperature for POPC (A) and POPC/POPS (B) vesicles, in the absence (black) and presence (color) of CPZ. The lower panels represent the hyperfine coupling (2A_Z_), used as a proxy for polarity, in POPC (C) and POPC/POPS vesicles (D) in the absence (black) and presence (color) of CPZ.

EPR spectra collected at room temperature carry information on the wobbling motion of lipid regions around the paramagnetic probe confined by the membrane. The local dynamics, represented by the ratio of the second over the third EPR line, h_0_/h (Supplemental Fig. S12B), provide insight on membrane fluidity: the higher the value, the stiffer the local membrane environment (66). In pure POPC vesicles, CPZ did not significantly change the order parameter regardless of the position of the spin probe, only subtly increasing rigidity at the 5-C position (Fig. 4A). Conversely, such 5-C position rigidification, corresponding to the upper acyl chain region, was much more pronounced when POPS lipids were present (Fig. 4B). This suggests that in the presence of POPS, CPZ hampers membrane motions near the 5-C position of the lipid molecules in the acyl chain region proximal to the headgroups, indicating a more specific interaction in this region of the bilayer with the anionic phospholipid.

To be able to compare the local polarity differences, EPR spectra were then measured after freezing the samples, thereby improving signal-to-noise ratio and minimizing molecular motions (37, 67, 68). Such “cryo” conditions allowed the extraction of the hyperfine coupling with the corresponding 2Az parameter (Supplemental Fig. S12A), which reflects the local polarity and hydration around the spin probe. Lower 2A_Z_ values correspond to a less hydrated environment (Fig. 4C–D, the scale is inversed). In POPC membranes (Fig. 4A, C), while the headgroup (T-PC) and 5-C position were slightly affected by CPZ, the more pronounced variation of 2Az was observed at the 10-C position, suggesting a decrease in polarity mostly in the membrane's center. A similar behavior, yet more marked, was observed in mixed vesicles containing POPS: the addition of CPZ triggers a significant decrease in polarity in the central part of the bilayer, whereas a slight increase is observed around the headgroup (Fig. 4D), indicating that CPZ reduces water content in the center of the membrane.

Altogether, these results suggest that CPZ addition affects the membrane environment in the tail region of the lipid bilayer, with a reduction in lipid mobility around the 5-C position and an overall decrease in polarity across the lipid bilayer hydrophobic region. Both these effects were shown to be enhanced by the presence of 10% POPS in the membrane.

#### All-atom molecular dynamics simulations

To complement the EPR data and gain an atomistic understanding of how CPZ affects lipid dynamics in a depth-dependent manner, we performed all-atom molecular dynamic (AA-MD) simulations. Unlike EPR, which probed the impact of CPZ solely on POPC, AA-MD simulations additionally measured the impact on POPS phospholipids, thus allowing us to disentangle the AP impact on separate phospholipids. Simulations quantify the C-H bond order parameter (S_CH_) along each acyl chain of both POPC and POPS, the saturated (palmitoyl) chain with 16 carbons (Sn2) and the monounsaturated (oleoyl) chain containing 18 carbons (Sn1) (69). The order parameter reflects the rotational dynamics around separate carbon atoms, where the higher value represents a more ordered environment (69).

**Fig. 5:**
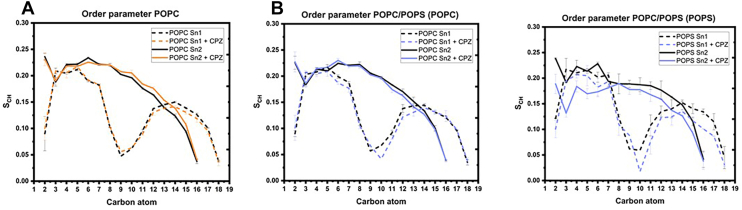
C-H bond order parameters (S_CH_) for each carbon atom along the Sn1 (C_1_-C_18_, oleoyl chain) and Sn2 (C_1_-C_16_, palmitoyl chain) of POPC in the different lipid model systems determined by AA-MD simulations. The panel (A) represents the POPC membrane in the absence (black) and presence of CPZ (orange) and the panel (B) POPC/POPS membrane in the absence (black) and presence of CPZ (blue). Sn1 acyl chain is represented with a dotted line (where the local S_CH_ reduction corresponds to a double bond presence) and Sn2 with a full line (70).

The impact of CPZ on POPC was minor in both simulated systems (Fig. 5A–B, first graph), consistent with observations on POPC membranes from EPR measurements. In contrast, the effect on POPS was substantially stronger (Fig. 5B, second graph), indicating a pronounced fluidification of the lipid chains.

### POPS preferentially interacts with CPZ and favors its less deep membrane insertion

The structural insights and atomistic simulations so far suggest that CPZ interacts and partitions within membranes in an anionic lipid-dependent manner. To quantify such preferential association/affinity, AA-MD simulations were used.

#### AA-MD simulations

**Fig. 6:**
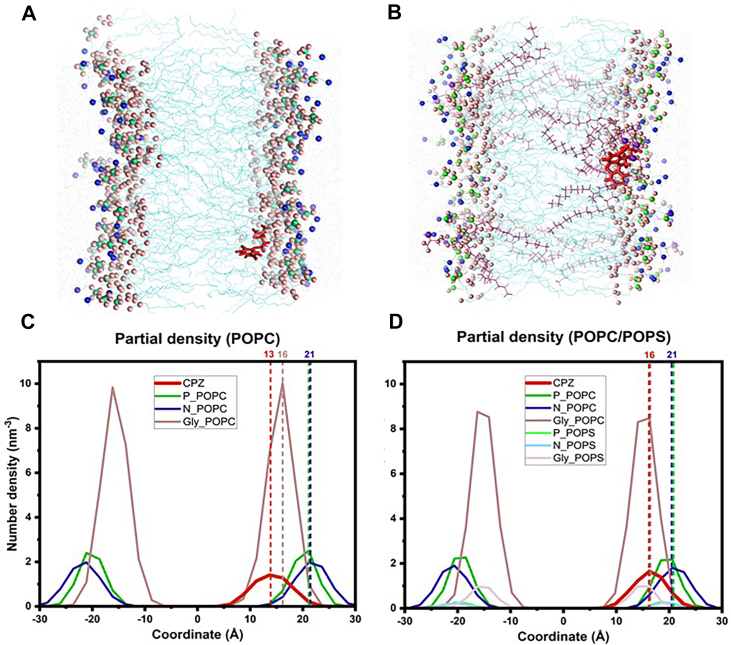
Representative snapshots (A, B) and normalized partial density profiles (C, D) of CPZ insertion in POPC and POPC/POPS bilayer obtained by AA-MD simulations. The snapshots from 500 ns show one CPZ molecule (red) inserted into a lipid bilayer composed of 100 POPC (A) or 90 POPC and 10 POPS lipid molecules (B). The POPS acyl chains are colored in purple. Normalized partial density profiles of CPZ and headgroup species along the bilayer normal (z) are shown for POPC (C) and POPC/POPS bilayer (C), where 0 represents the center of the bilayer. Glycerol oxygens are depicted in purple, nitrogen atoms in blue and phosphate atoms in green, with darker colors depicting POPC and lighter ones POPS species.

To further quantify the preferential localization of CPZ, normalized density profiles along the membrane normal were calculated from the AA-MD simulations, representing the distribution of AP relative to different phospholipid headgroup species (P, N, and glycerol) (71, 72). In a POPC bilayer, CPZ exhibits a broad distribution with significant penetration into the hydrophobic core, consistent with deep partitioning of amphiphilic drugs into neutral membranes, centered around 13 Å from the bilayer center (Fig. 6A, C). In contrast, incorporation of 10% POPS results in a redistribution of CPZ density towards the more interfacial region of around 3 Å (16 Å from the center), with a decreased penetration depth (Fig. 6B).

**Fig. 7:**
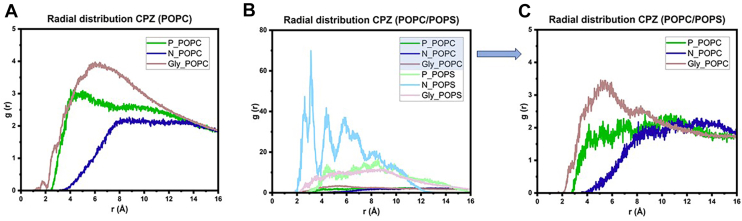
Radial distribution of N and P atoms and glycerol group of phospholipids around CPZ center of mass. The panel (A) represents a POPC bilayer and the panel (B) a POPC/POPS bilayer. Panel (B) represents the distributions of both POPC and POPS phospholipid species, and panel (C) the distribution of POPC species on the same bilayer, where the scale is modified.

To better understand how CPZ interacts with specific headgroup regions of POPC and POPS lipids and to determine the distance and extent of these interactions, the RDFs (g(*r*)) computed between CPZ and phosphate (P), choline (N), and glycerol oxygen (Gly) were compared. In the pure POPC system, the g(r) profiles reveal broad and well-defined peaks for P, N, and Gly regions, centered at 4, 8, and 6 Å from the CPZ, respectively, with the maximal intensity observed for Gly species (Fig. 7A). This suggests that CPZ is predicted to insert partially into the membrane, establishing contacts with the upper part of the lipids while remaining anchored via polar headgroup interactions (Fig. 7A). In the mixed bilayer composed of POPC and POPS, the overall shape of RDFs for POPC headgroups remains similar (Fig. 7B, second graph). However, and interestingly, new and distinctive peaks appear for the POPS functional groups, particularly N_POPS. This group exhibits a sharper and more intense peak at around 3 Å, noticeably more localized than its N_POPC counterpart (Fig. 7B, first graph). This suggests that the amine group of POPS interacts with CPZ in a more specific and spatially constrained manner, likely because of favorable electrostatic or hydrogen bonding interactions. The g(*r*) for P_POPS and Gly_POPS was broader and less intense, implying less specific interactions (Fig. 7B, first graph). Overall, RDF analysis provided atomistic evidence for preferential and specific interactions between CPZ and POPS headgroups, consistent with enhanced CPZ retention and interfacial localization observed experimentally by NR and EPR.

## Discussion

Understanding how APs interact with biological membranes is crucial, as their accumulation in the lipid bilayers likely underlies both the delayed onset of full clinical effects and their prolonged activity observed after treatment discontinuation (9). Among these APs, CPZ is of particular interest because of its ability to enhance POPS synthesis (24). To better dissect how negatively charged lipids influence CPZ/membrane interactions, we combined complementary biophysical and computational approaches on two simplified model systems: pure POPC and a mixed POPC/POPS (9/1) membrane.

Fluorescence spectroscopy first showed the strong membrane partitioning of CPZ, consistent with its high lipophilicity (logP = 5.18). The inclusion of 10% POPS significantly enhanced AP partitioning, suggesting stronger incorporation of CPZ into the membrane, possibly through electrostatic interactions between the positively charged amine group of CPZ and the negatively charged POPS headgroups. NR experiments confirmed CPZ insertion throughout both bilayers in comparable amounts, yet with significantly higher retention in the POPC/POPS model system after rinsing with buffer. AA-MD simulations predicted that CPZ only penetrates superficially in the bilayer—remaining near the glycerol region in all simulations, an effect that was more pronounced in the presence of POPS compared with the slightly deeper insertion in pure POPC membranes. RDF analyses additionally, and consistently, highlighted short-range electrostatic interactions, particularly between CPZ amine and the negatively charged POPS, especially next to -COO^-^ groups, confirming specific lipid-drug interactions that promote the drug’s preferential anchoring closer to the bilayer surface in the simulations. In contrast, POPC exhibited much weaker specific interactions with CPZ. Although speculative because of the higher estimated uncertainties, the NR results on the accumulation of CPZ in the headgroup regions of the POPS-containing bilayer align with the AA-MD simulations. These findings align well with solid-state NMR and fluorescence studies demonstrating preferential CPZ binding and affinity to PS headgroups (26, 73, 74).

Markedly, AFM and NR measurements demonstrated that such CPZ partitioning/insertion leads to membrane restructuring. Both techniques indeed reveal that high CPZ concentrations induce an overall membrane thinning, regardless of the lipid composition: ∼9 Å thinning for POPC and ∼4 Å for POPC/POPS probed by AFM at 500 μM CPZ, whereas NR reported similar thickness reductions of ∼7 Å (POPC) and ∼9 Å (POPC/POPS). AA-MD simulations corroborated these findings, yet with more subtle thinning effects (0.2–1.7 Å, Supplemental Table S5), likely because of the use of a single CPZ molecule per simulation. The observed membrane thinning also correlated with a slight increase in average area per lipid observed in AP presence, as expected. Though the standard deviations are large, the same tendency is observed across all the systems studied (Supplemental Table S5). Such effects are favored when increasing CPZ-to-lipid ratio (from 1/100 to 1/25) and enhanced in the presence of anionic POPS lipids. These findings are consistent with *i*) studies reporting an inverse relationship between both properties and *ii*) CPZ fluidifying effect previously hypothesized as it decreases the phase transition temperature for dipalmitoylphosphatidylcholine (75, 76, 77, 78).

Interestingly, at high CPZ concentrations (50 and 500 μM), above the critical micelle concentration (∼20 μM), POPC/POPS bilayers occasionally exhibited a two-step breakthrough behavior that was never observed at lower CPZ concentrations or in pure POPC bilayers (19, 20). Such two-step rupture events have been previously and only reported in cholesterol-containing membranes supported on mica, in which high cholesterol triggers interleaflet decoupling and subsequently a layered architecture with distinct mechanical signatures in AFM-based spectroscopy (79, 80). Our observations thus indicate the presence of two mechanically distinct layers upon CPZ interaction, and only when anionic lipids are present—possibly an upper CPZ-lipid aggregate layer and the SLB. NR experiments provided complementary and noncontact evidence for this hypothesis, as they identified a diffuse adsorbed layer above both POPC and POPC/POPS bilayers, composed of lipid-CPZ aggregates. While in POPC, this interfacial layer appeared broadly distributed up to 124 Å above SLB; in POPC/POPS membranes, it only extended up to ∼61 Å, likely connected to the SLB via low-density “stalk” structures. The combined AFM and NR data thus point toward the formation of lipid-CPZ aggregates above the bilayers, as previously reported at AP concentrations above 50 μM (81). This is consistent with previous EPR experiments revealing mixed micelle formation at high CPZ/lipid ratios and simulations, where detergent presence promoted the formation of worm-like micelles (82, 83).

These structures might be stabilized by anionic lipids through long-range electrostatic interactions or via “stalks,” transient hourglass lipid structures that connect outer leaflets of two separate bilayers. Stalks, as membrane fusion intermediate structures, have been observed and energetically characterized previously (84, 85, 86). Moreover, during lamellar-to-nonlamellar phase transitions, lipid membranes can spontaneously form stalk-like interbilayer connections that act as curvature-driven topological intermediates and may exist as transient fluctuations or even stable rhombohedral stalk phases (87, 88, 89). The proposed “stalk” formation is in agreement with prior studies reporting that membrane dehydration represents a key factor for promoting stalk formation, consistent with the fact that POPS-containing bilayers provoke a more prominent bilayer dehydration of the center (86). Additionally, Chol promotes the stalk formation, and it has been previously proposed that APs, notably CPZ, may display a “Chol-like” effect on the bilayers (78, 86). Such stabilization might explain why the two-step breakthrough behavior is only observed in POPS-containing systems by AFM, unlike in NR: the AFM tip first penetrates stable micellar or bicellar aggregates and then the underlying SLB.

The membrane structural rearrangement induced by CPZ and promoted by POPS goes with alterations in membrane ordering, polarity, and phospholipid mobility. While Laurdan fluorescence spectroscopy indicated increased rigidity in the headgroup/glycerol region for both POPC and POPC/POPS systems, EPR spectroscopy provided higher spatial resolution and revealed a pronounced rigidification near the 5-C position and reduced polarity in the bilayer center upon CPZ addition. These results were significantly stronger in POPS-containing membranes and reveal a stronger order-inducing interaction of the drug with PS-containing membranes. AA-MD simulations further validated these findings by showing preferential interactions of CPZ with POPS, leading to localized ordering of the lipid headgroups and proximal acyl chain regions. Together, these data indicate that CPZ rigidifies the outer bilayer regions also previously reported by EPR (90). These results complement previous fluorescence studies showing that CPZ affects both membrane headgroups and tail regions (91). Besides, those effects appear to be a more general effect of amphiphilic APs rather than molecule-specific phenomena, as similar behavior was reported for other neuroleptics—olanzapine and thioridazine—particularly in the presence of negatively charged phosphatidylglycerol lipids (68).

**Fig. 8:**
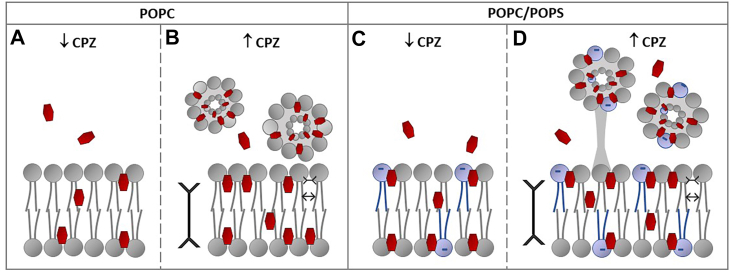
A schematic representation of a proposed model for CPZ’s interaction with POPC (A, B) or POPC/POPS bilayer (C, D) at low (A, C) and high concentrations (B, D). At low CPZ concentration in the POPC bilayer (below CMC), the molecule inserts in the membrane, preferentially around the glycerol level (A). At a higher concentration (above CMC), the AP presence leads to membrane thinning and increased headgroup ordering. Mixed micelles are present above the bilayer (B). In the POPC/POPS bilayer, CPZ preferentially inserts a bit higher—around the phosphate/glycerol level (C). When CPZ is present in high concentrations, the impact on the bilayer itself is similar to POPC-only bilayer, with stronger membrane partitioning and stronger dehydration of the center of the bilayer. Above the membrane, mixed micelles composed of lipids and CPZ are present and may be connected to the bilayer by thin lipid structures: “stalks,” which stabilize the micelles (D, represented in gray).

Taking all the data into account, our results demonstrate that CPZ interacts with phospholipid bilayers in a concentration-dependent manner, with distinct effects observed in the presence of anionic PS lipids. Structural analyses reveal that high CPZ concentrations provoke bilayer thinning and rigidification around the headgroup regions, both in PC-only and PC/PS membranes. Moreover, mixed micelles form above the bilayer (Fig. 8B, D). In the presence of PS, the drug is preferentially localized a bit more superficially—at the phosphate/glycerol level compared with the PC-only bilayer (glycerol level). The preferential interactions between PS and CPZ enhance its membrane partitioning and promote greater retention of the drug after the removal of excess CPZ from the system. These interactions also decrease the mobility at the beginning of the acyl chains and lead to a greater overall polarity reduction within the hydrophobic core of anionic lipid bilayers. Mixed micelles observed are possibly connected to the bilayer with “stalks,” and the electrostatic forces play a crucial role in their stabilization (Fig. 8D).

To conclude, these results underscore the critical role of membrane charge in governing the partitioning and localization of amphiphilic drugs within lipid bilayers as well as their preferential association with negatively charged lipids. Overall, POPS enhances CPZ membrane partitioning and results in a stronger dehydration of the bilayer core and a more distant, stable distribution of mixed micelles. Such differential interactions of CPZ with specific lipid environments can be related to physiological contexts, as alterations in POPS content have been reported in membranes of schizophrenic patients—both as a pathological feature and as a result of CPZ treatment (7, 28). In healthy eukaryotic cells, PS is located almost exclusively in the inner leaflet of the plasma membrane (92). Despite the fact that abnormal transbilayer distribution of phospholipids, like PE, has been observed in patients suffering from schizophrenia, few studies on AP-induced alteration in PS have addressed whether PS gets externalized to the outer leaflet (93). One report indicated that the incubation of erythrocytes with CPZ increases PS exposure in the outer leaflet, a redistribution that could lead to higher red blood cell death rates (7, 25). Clozapine, another AP, has been shown to increase reactive oxygen species and markers of apoptosis in cultured cells (94). PS externalization being a signature of apoptosis, this opens the question of whether this AP leads to PS externalization. Changes in lipid composition, independent of asymmetry aspects, and in CPZ-lipid interactions may directly influence the conformation and functionality of D2 and other receptors, either through direct binding or indirectly via altered membrane accumulation profiles and subsequent changes in the lipid membrane properties (29). PS modulation of G protein-coupled receptor activity in terms of ligand-induced conformational states and triggered intracellular signaling cascades, including the D2R, has been reported (34, 95, 96, 97, 98). Therefore, an increase in PS levels in the intracellular leaflet as a result of CPZ treatment can modulate the activity of critical effectors in intracellular signaling cascades orchestrated by G protein-coupled receptors, like the D2R. This, in turn, could modulate treatment efficacy and contribute to APs’ side effects. Understanding how APs partition and remodel membranes—as demonstrated here—thus offers valuable insight for drug design. Our study highlights the importance of considering not only the direct receptor-drug interactions but also the drug’s impact on membrane organization and dynamics.

## Data availability

All data and further information are available upon request to the corresponding authors.

## Supplemental data

This article contains supplemental data.

## Conflict of interest

The authors declare that they have no conflicts of interest with the contents of this article.
