# Supplementary figures and images for: The impact of charge on chlorpromazine interaction with lipid membranes
Source: J Lipid Res. 2026 Apr 6;67(5):101035. doi: 10.1016/j.jlr.2026.101035 (PMC13158409; doi:10.1016/j.jlr.2026.101035)

POPC

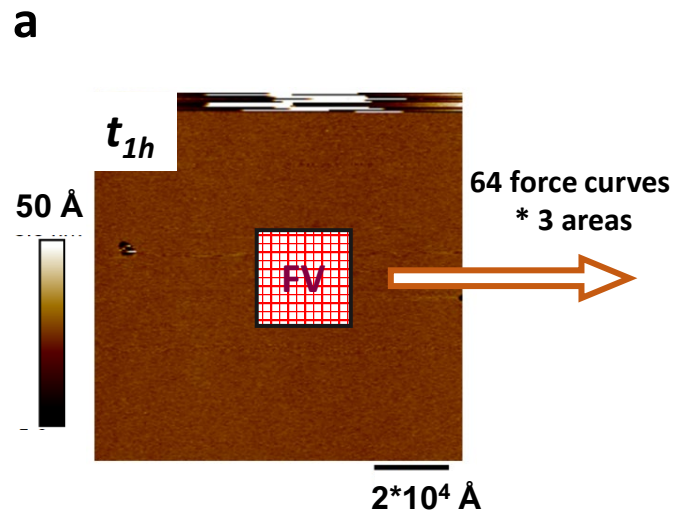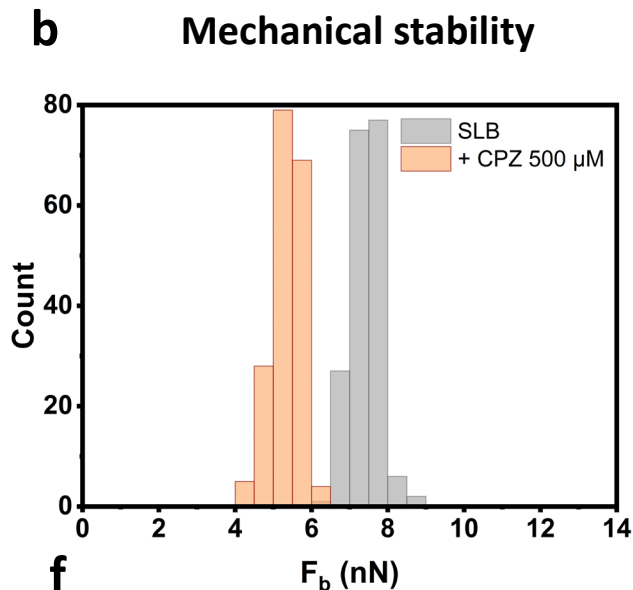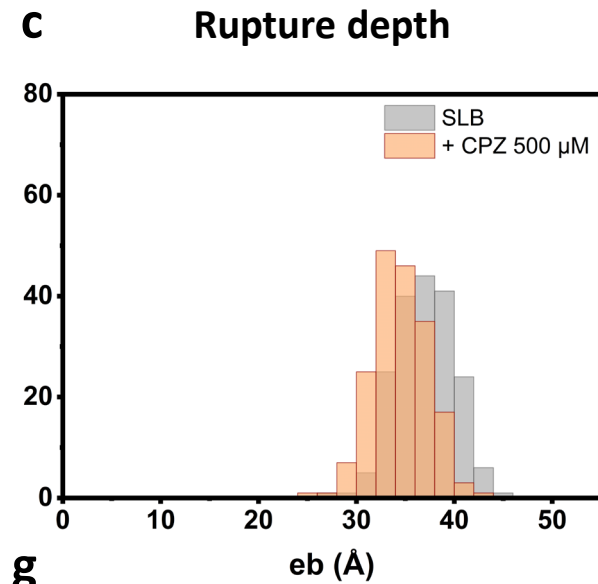

POPC/POPS

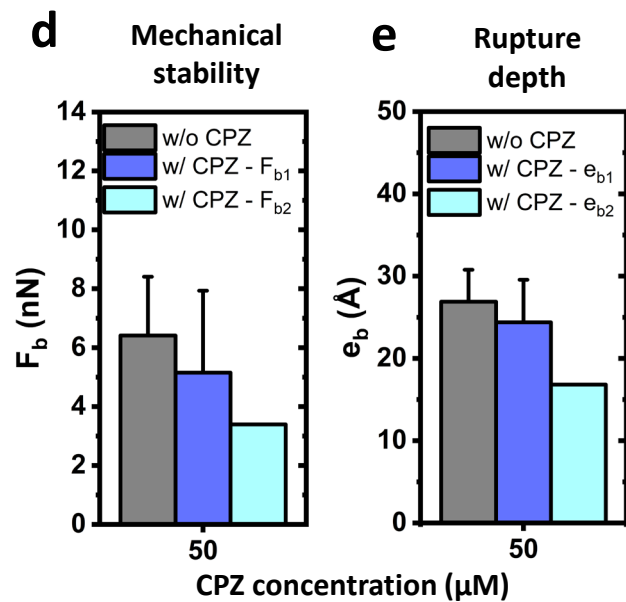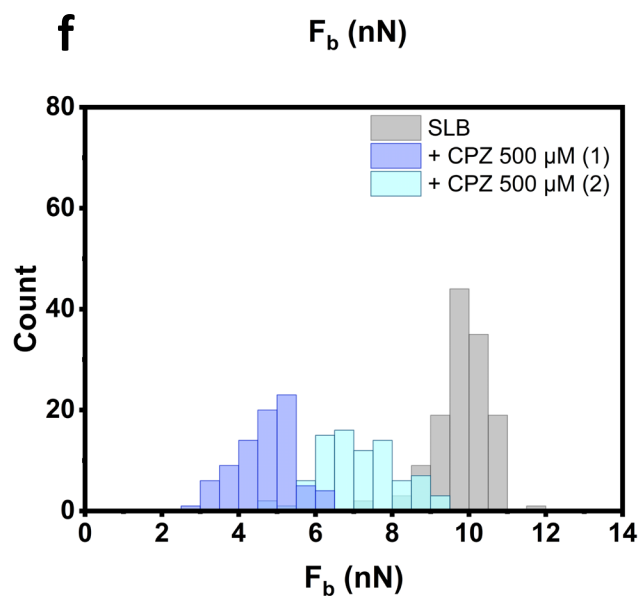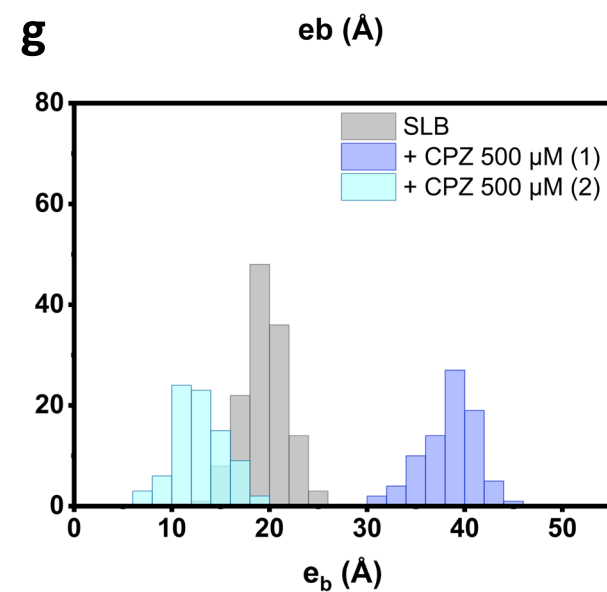

Supplement: Supplemental Fig 1 [file mmc1.pdf]

# POPC + 10 $\mu$ M CPZ

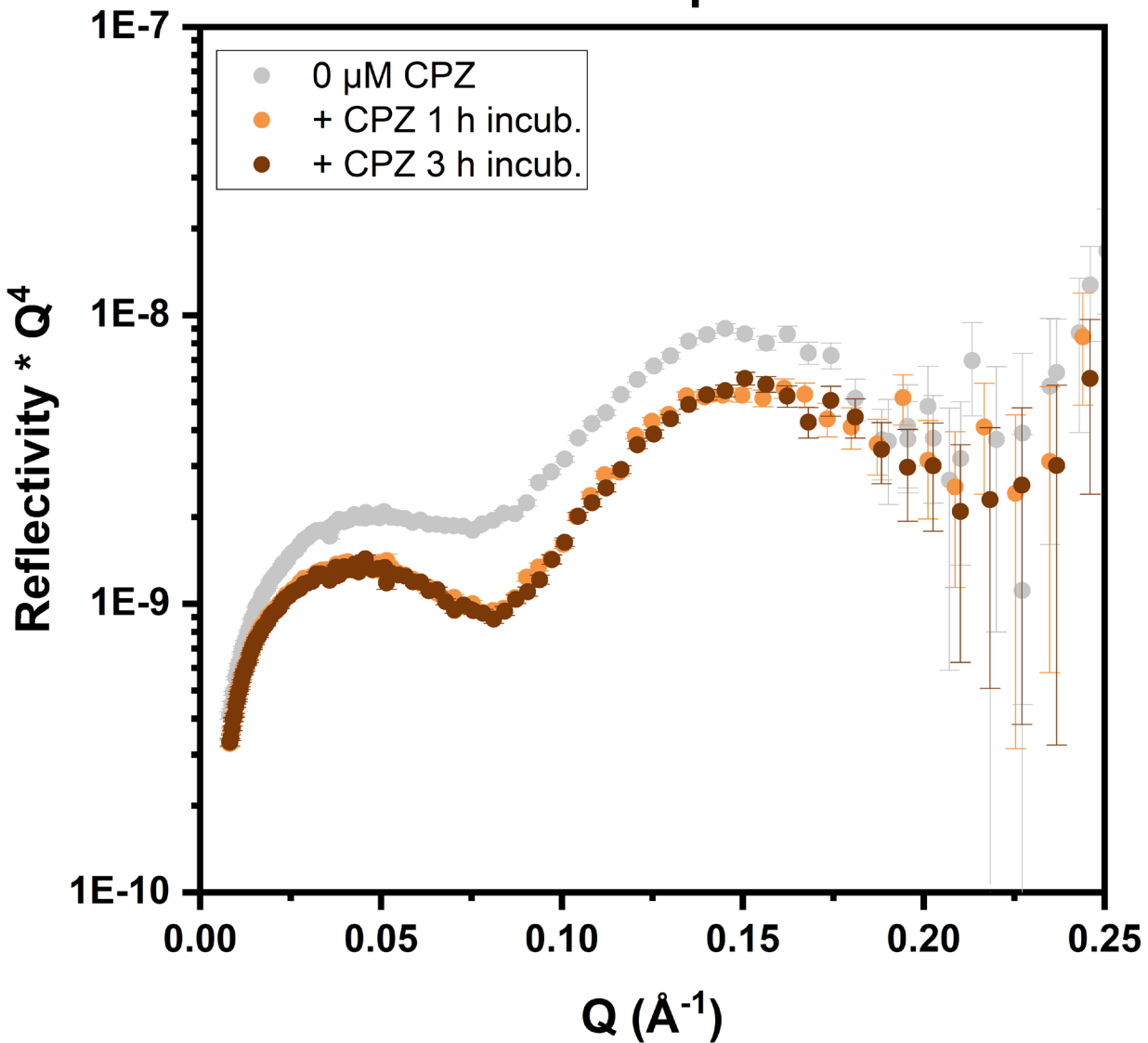

Supplement: Supplemental Fig 2 [file mmc2.pdf]

POPC Data and Fits

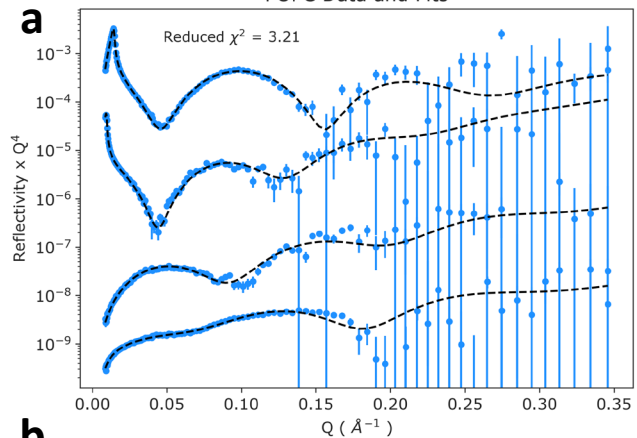

POPC SLD Profiles

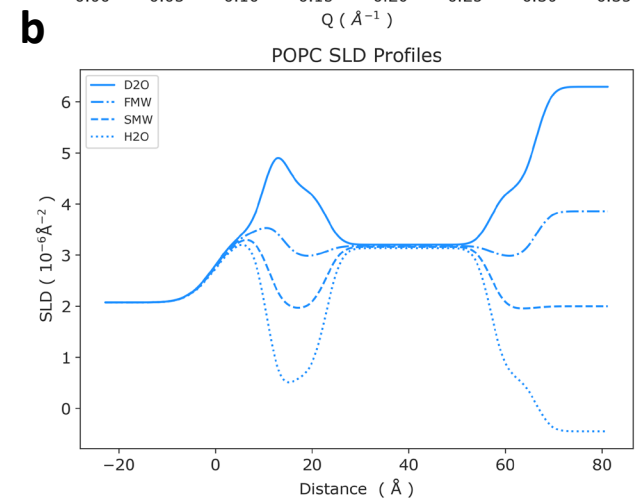

POPC VF

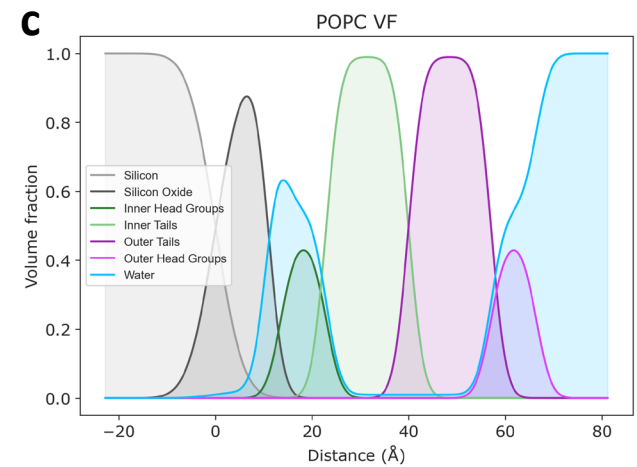**d**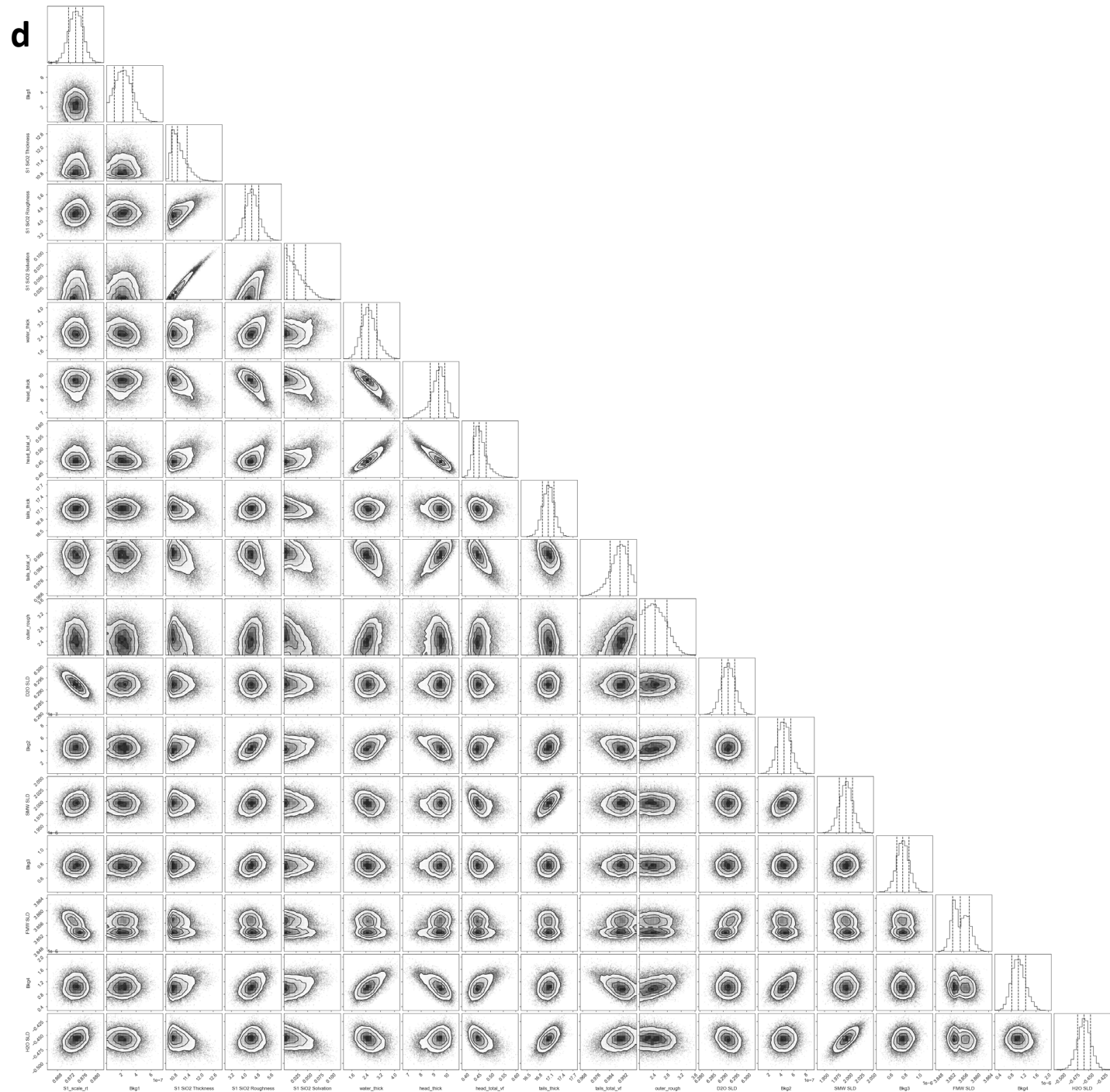

Supplement: Supplemental Fig 3 [file mmc3.pdf]

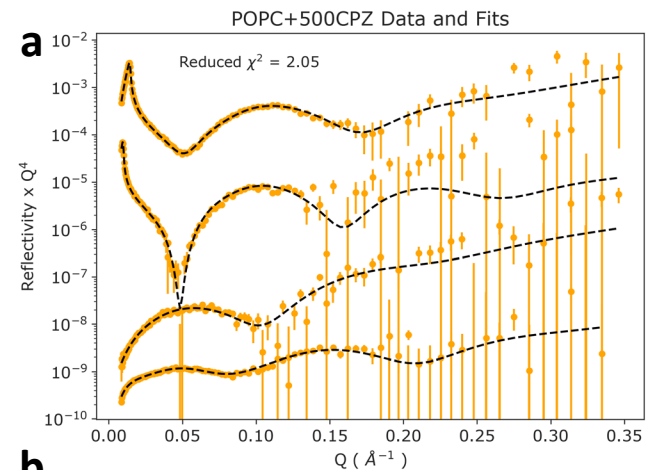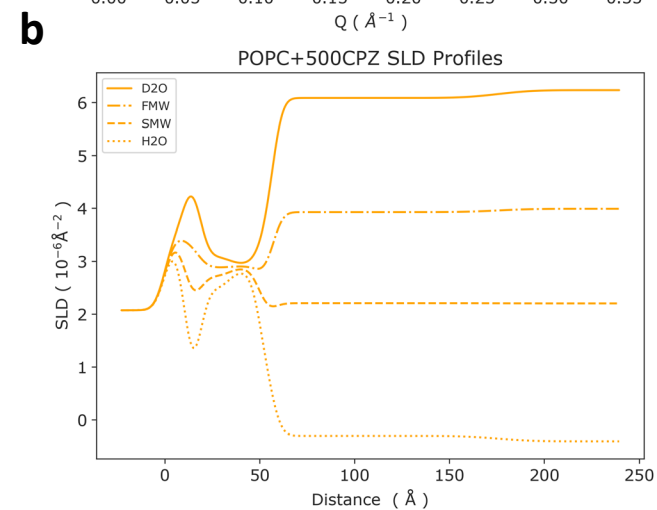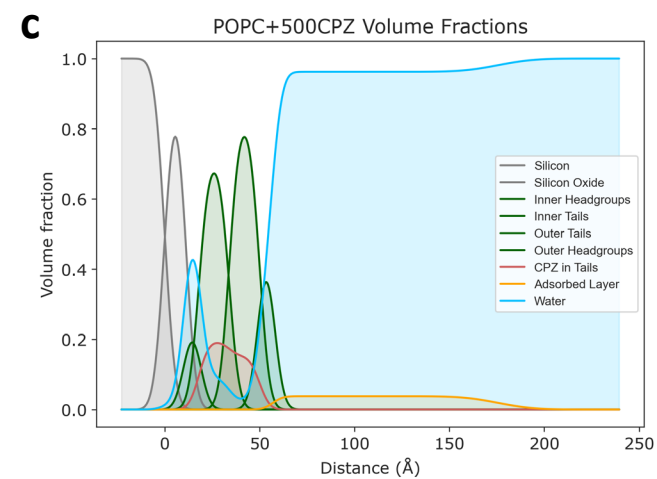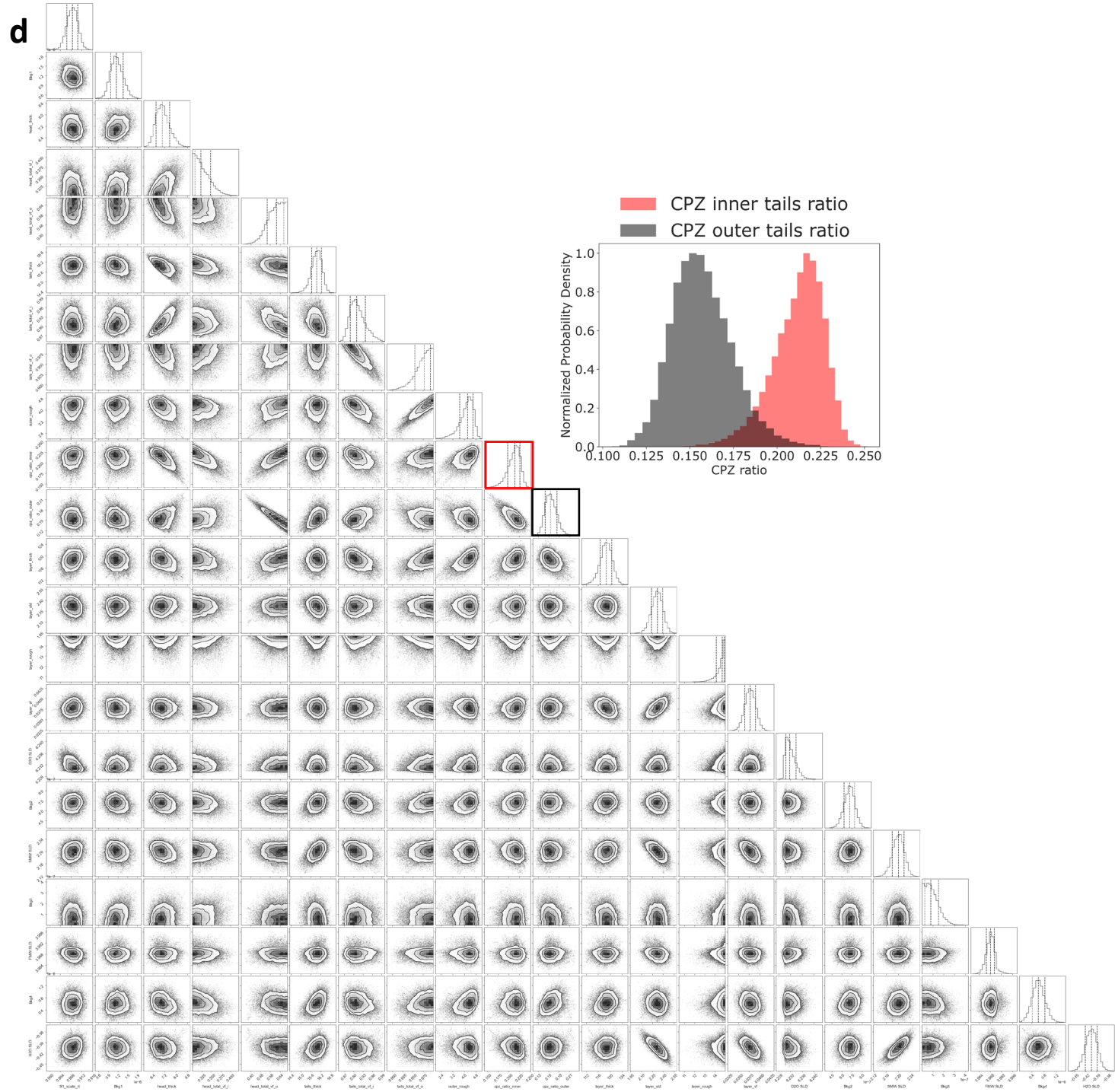

Supplement: Supplemental Fig 4 [file mmc4.pdf]

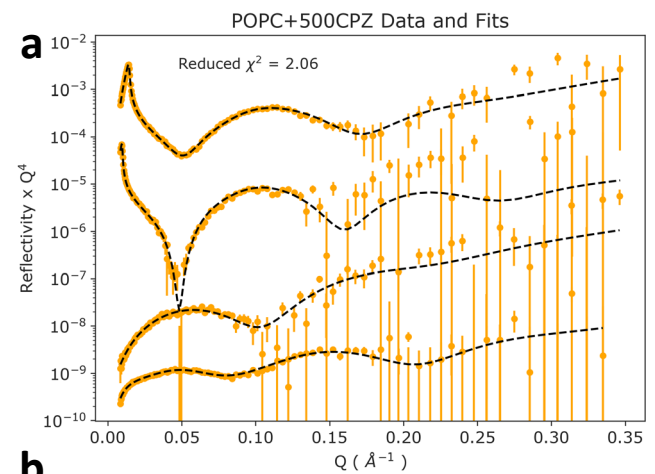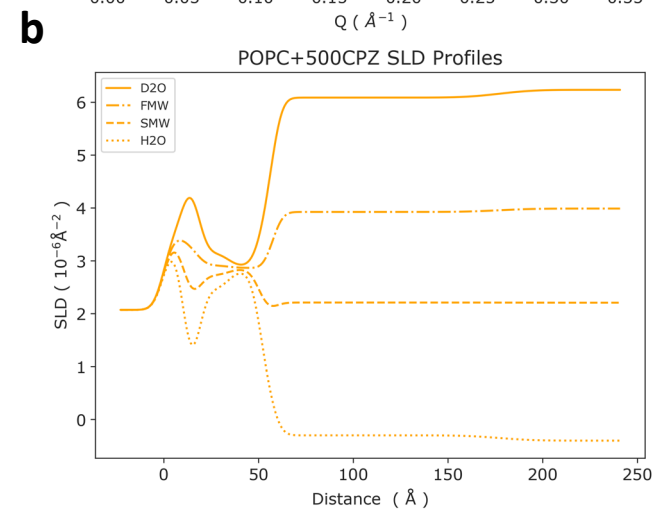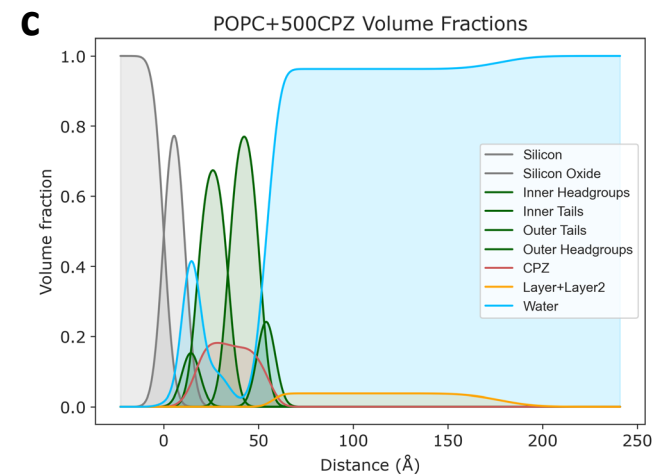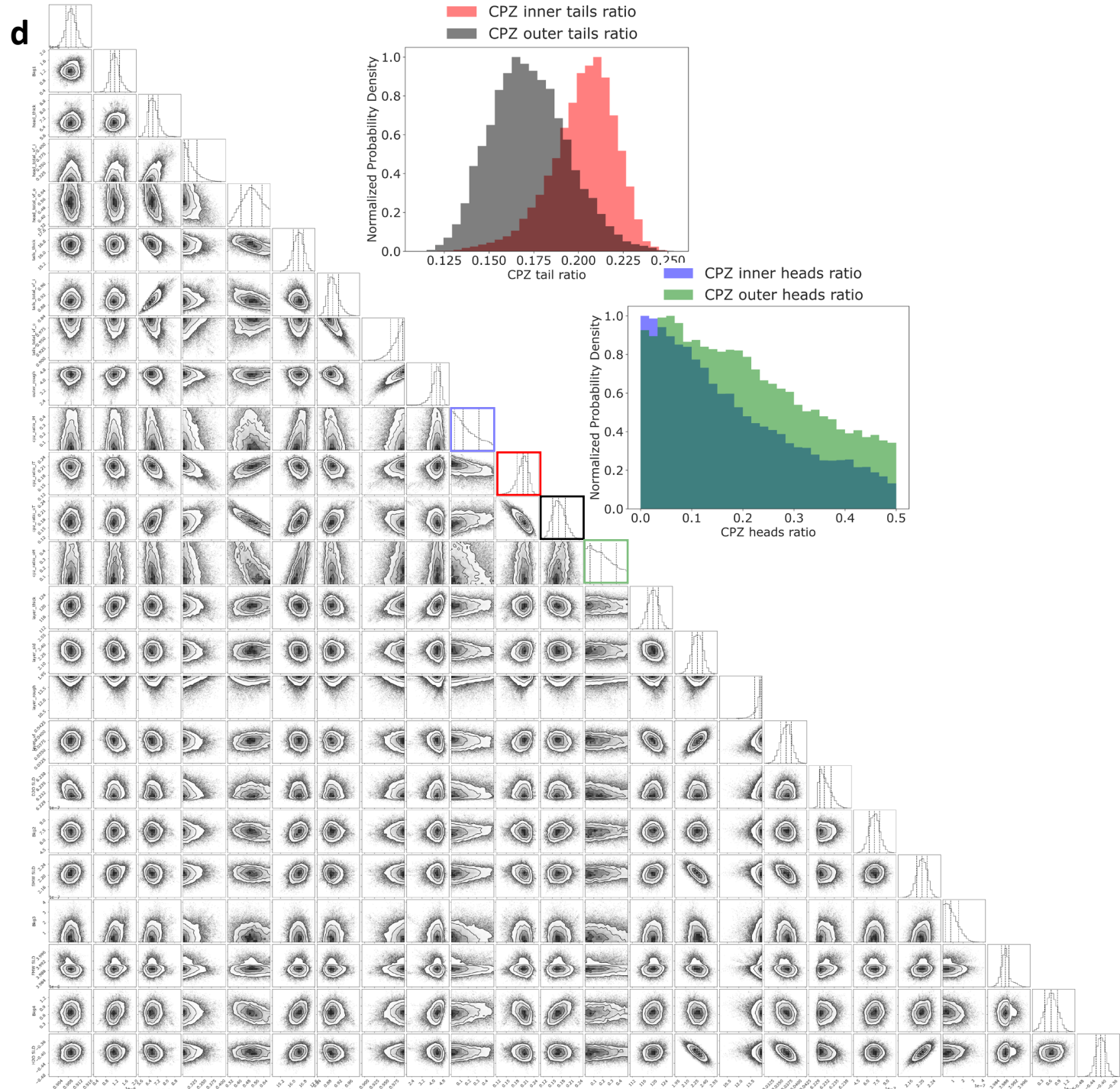

Supplement: Supplemental Fig 5 [file mmc5.pdf]

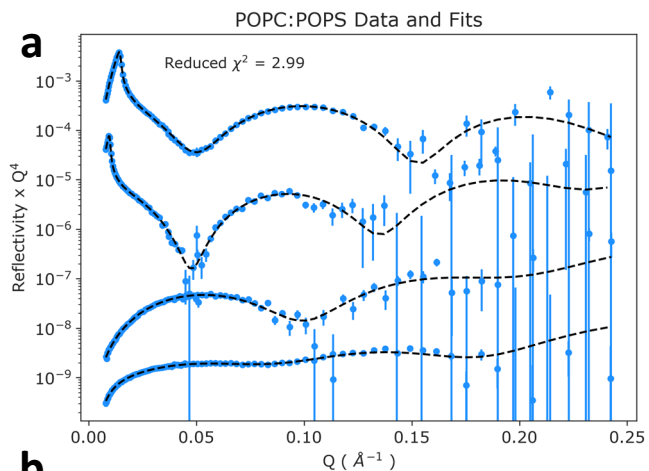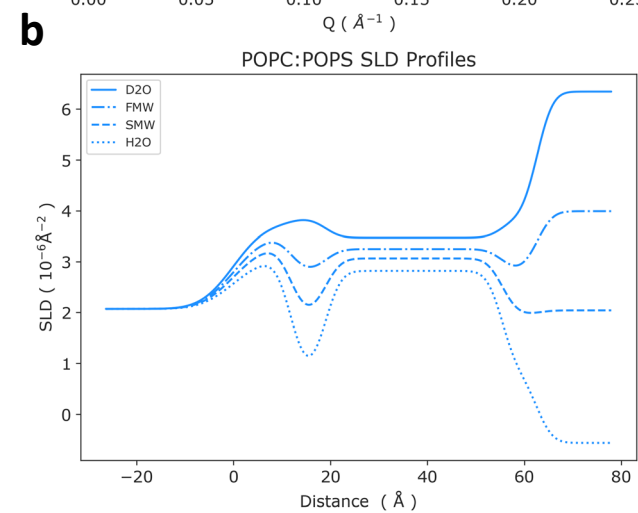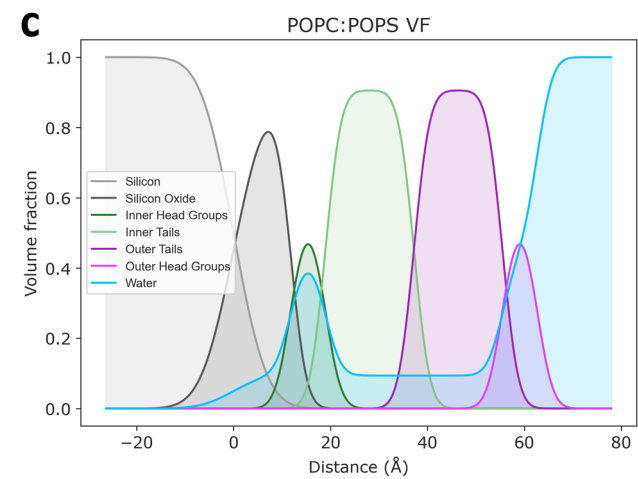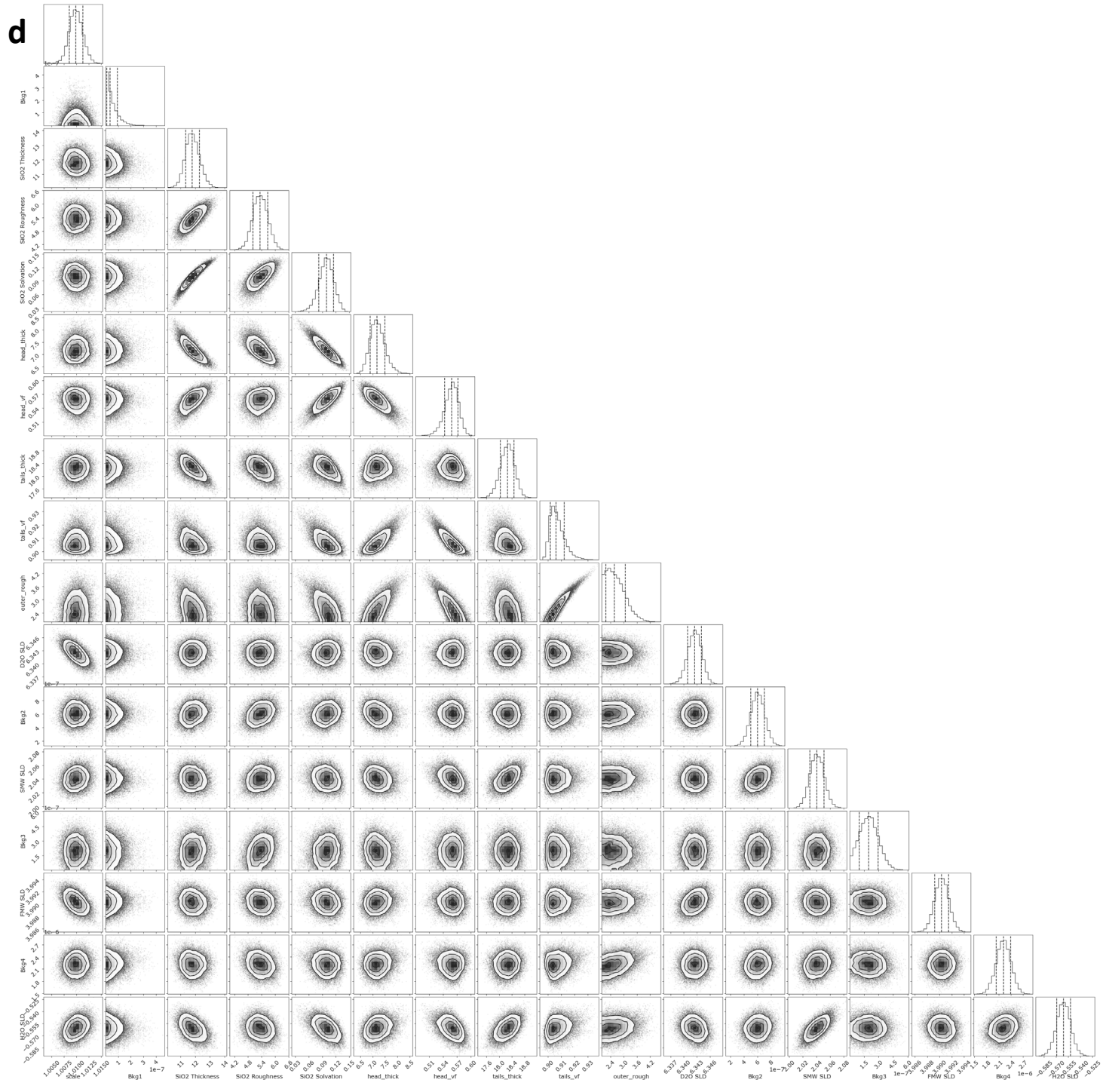

Supplement: Supplemental Fig 7 [file mmc7.pdf]

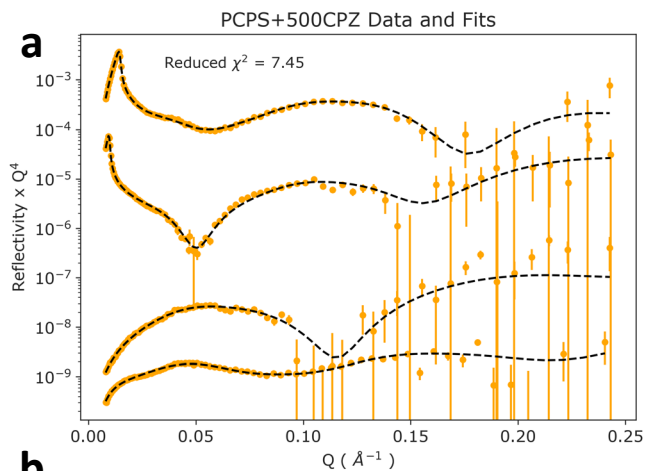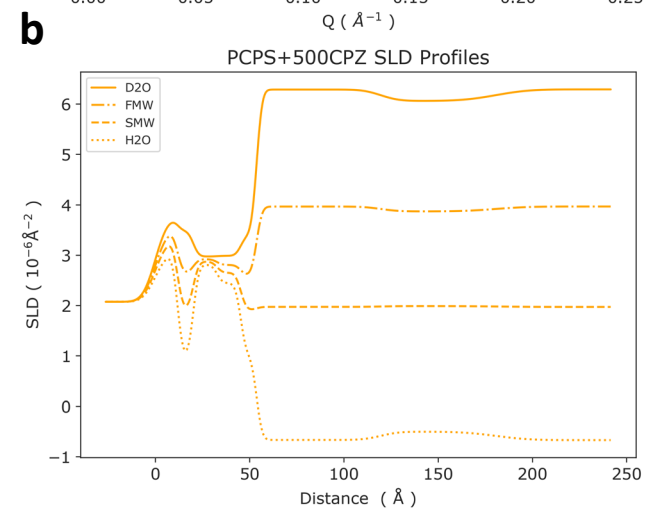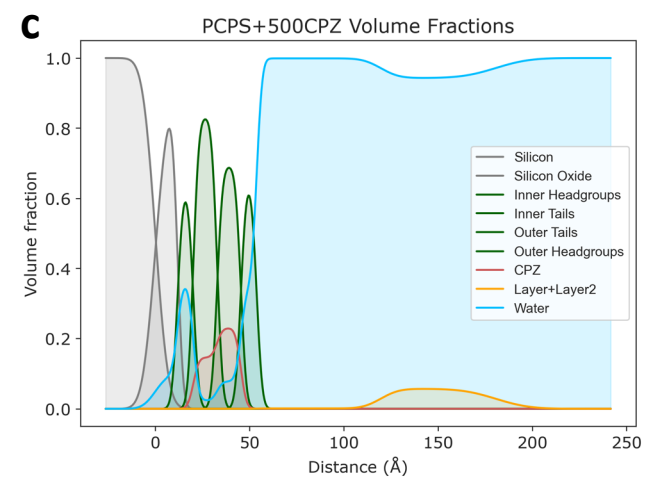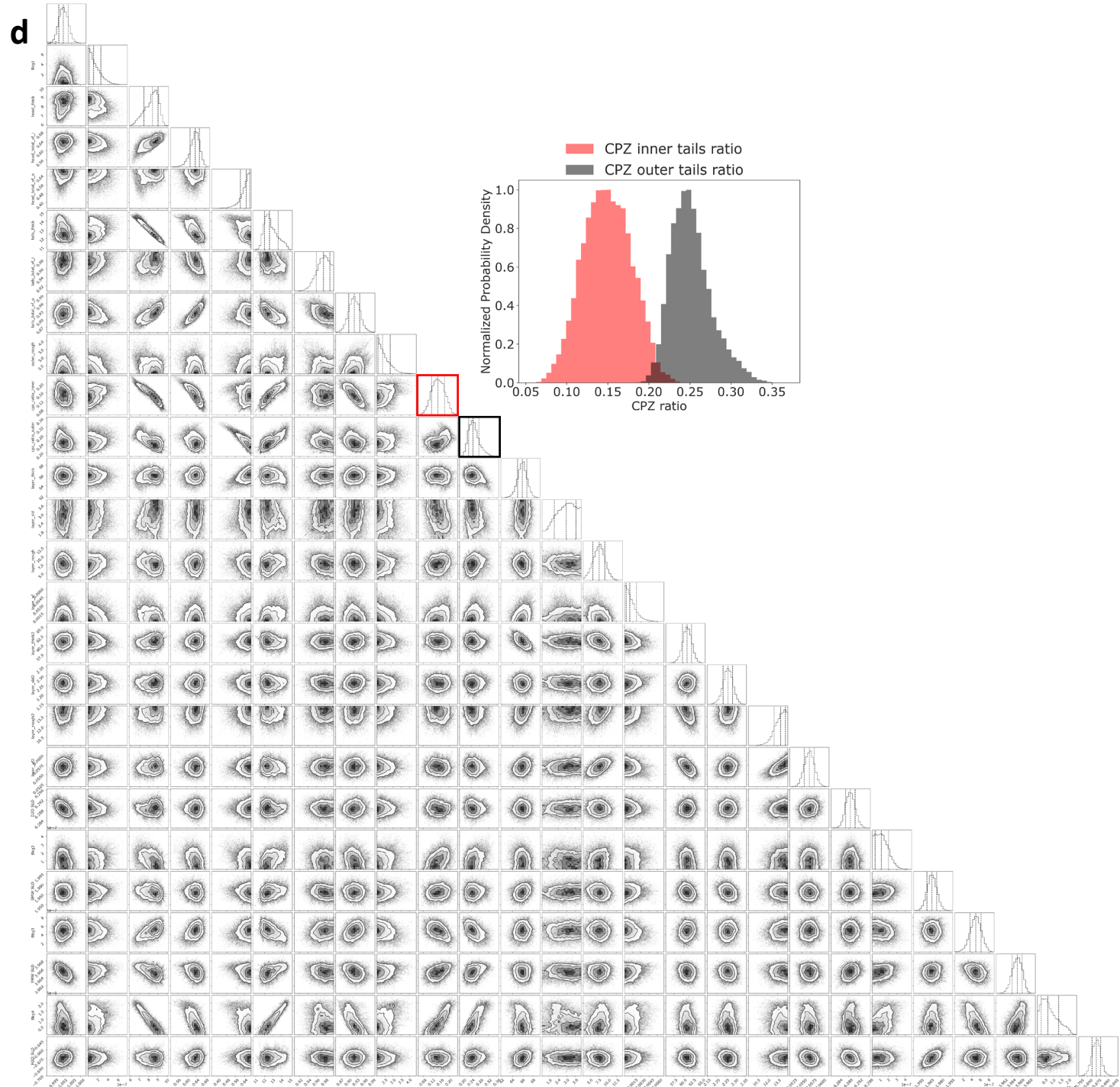

Supplement: Supplemental Fig 8 [file mmc8.pdf]

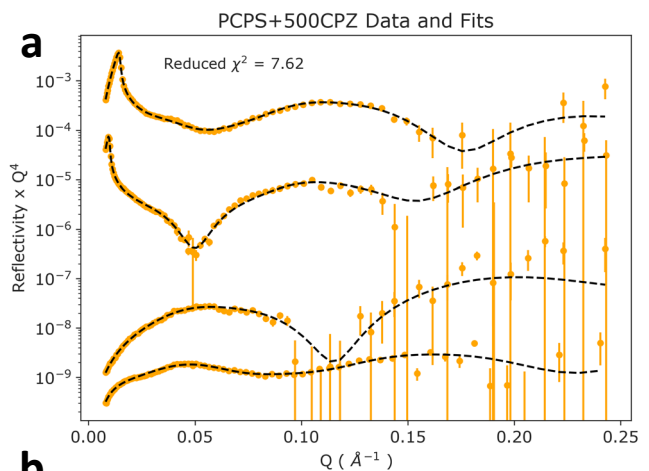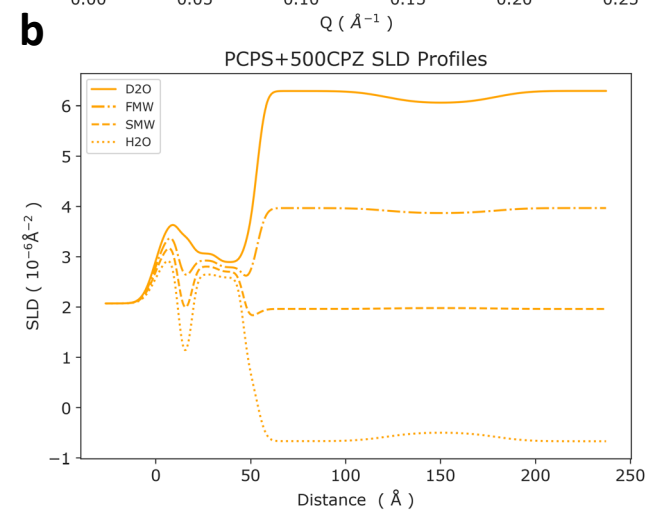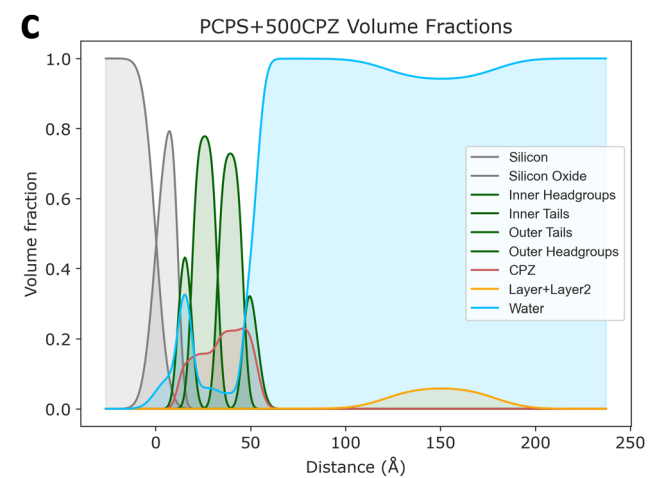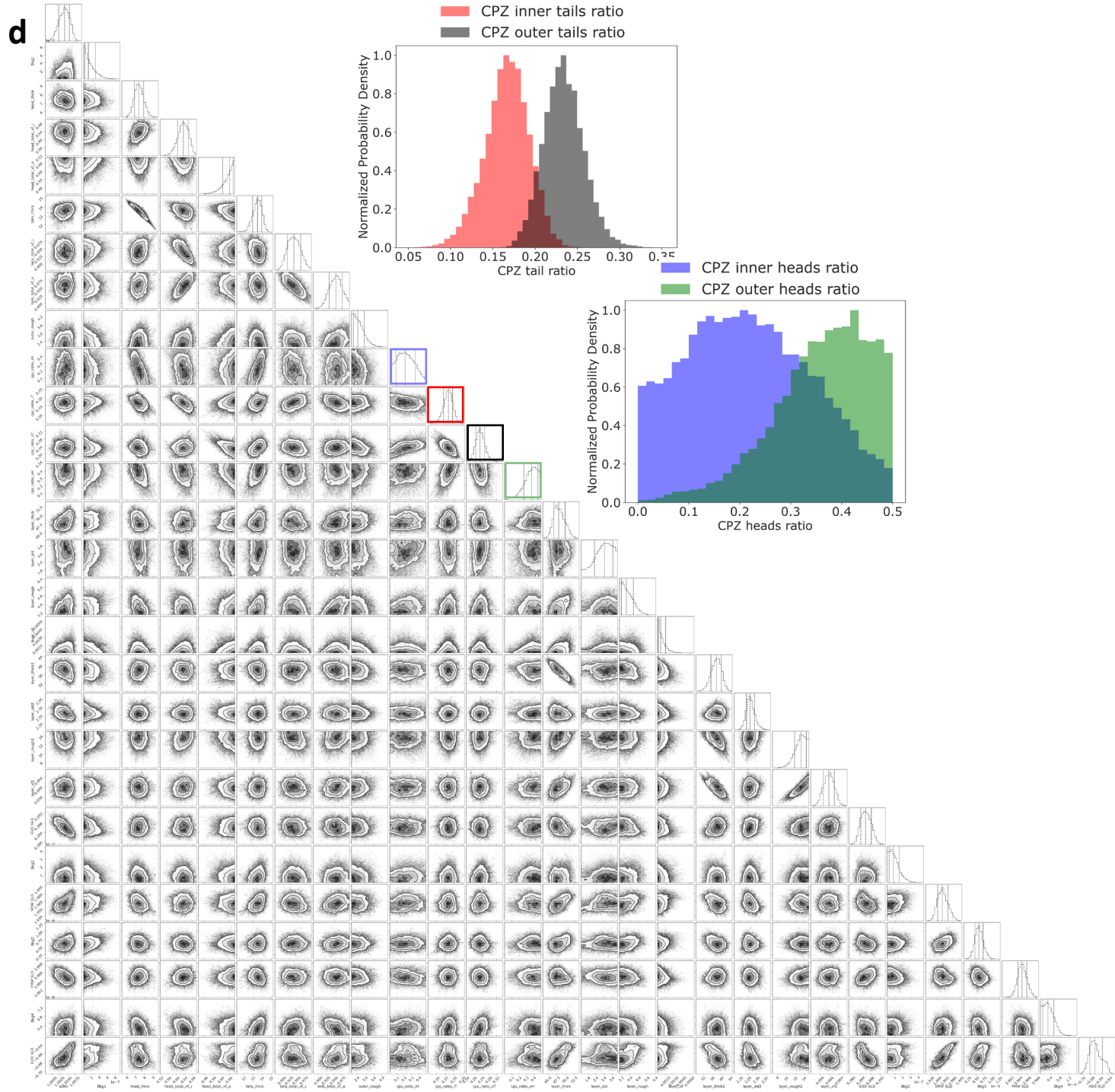

Supplement: Supplemental Fig 9 [file mmc9.pdf]

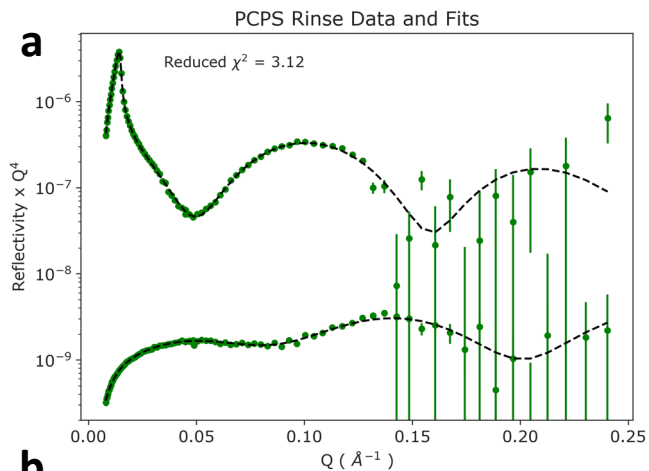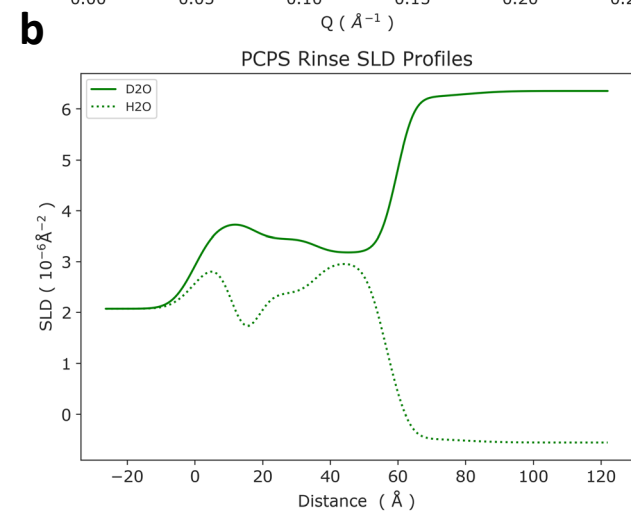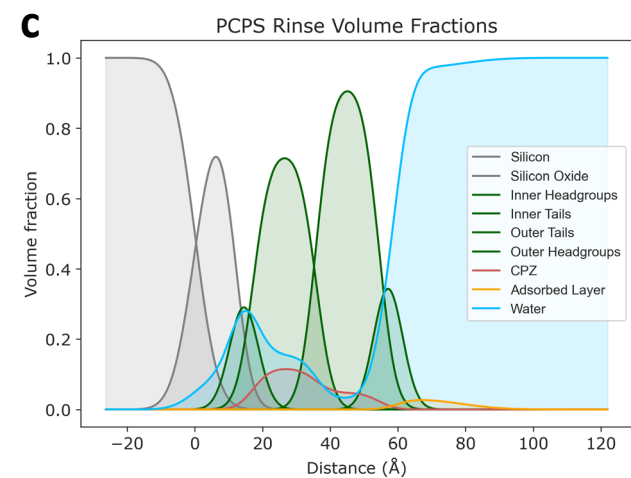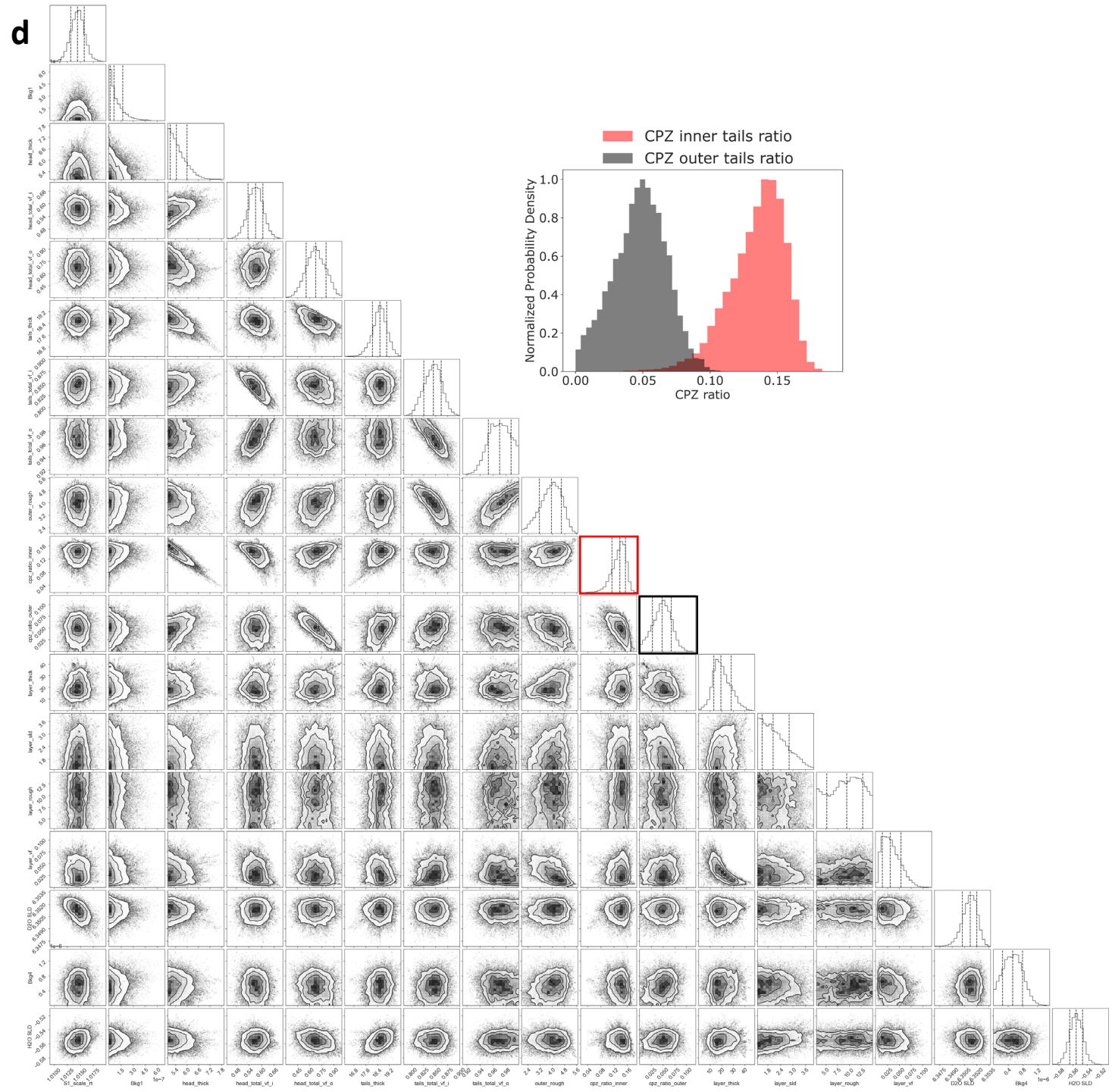

Supplement: Supplemental Fig 10 [file mmc10.pdf]

**a**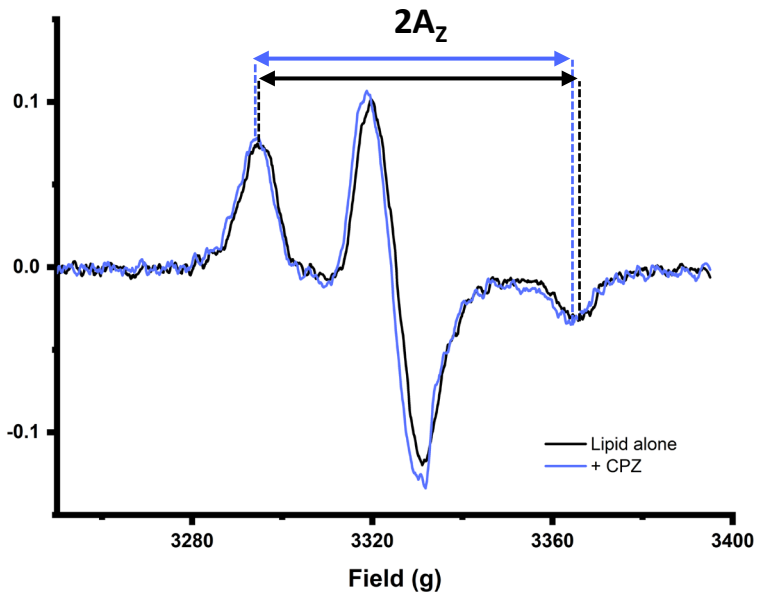**b**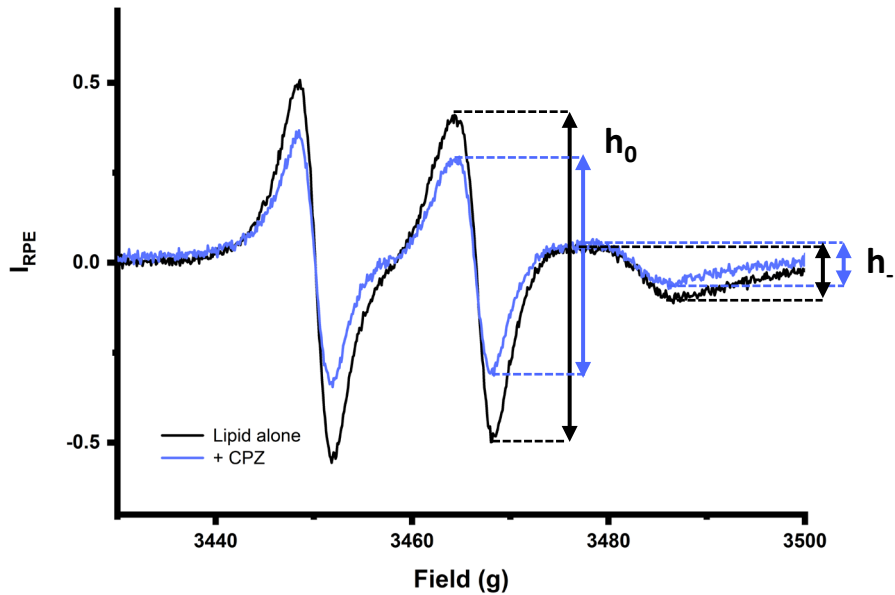

Supplement: Supplemental Fig 12 [file mmc12.pdf]

**a**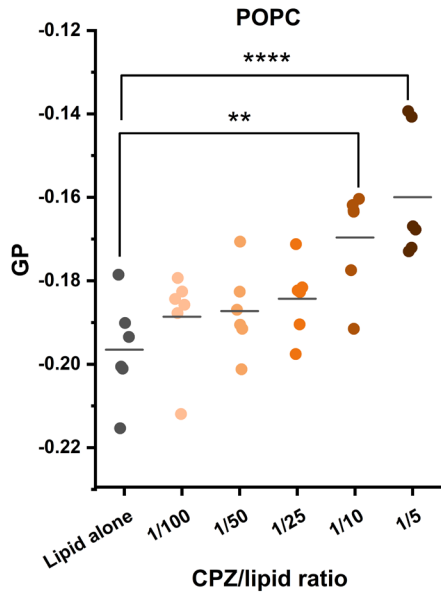**b**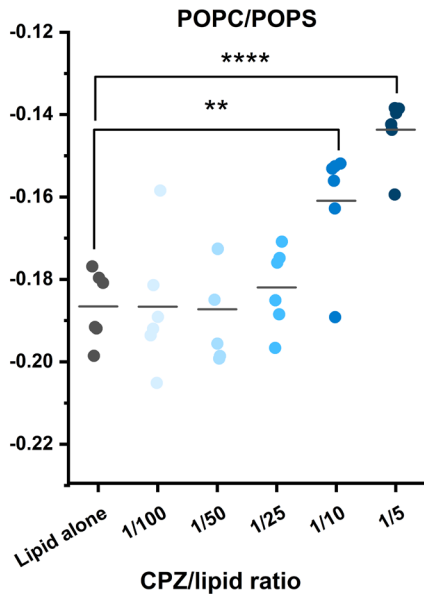

Supplement: Supplemental Fig 13 [file mmc13.pdf]

# CPZ fluorescence emission intensity

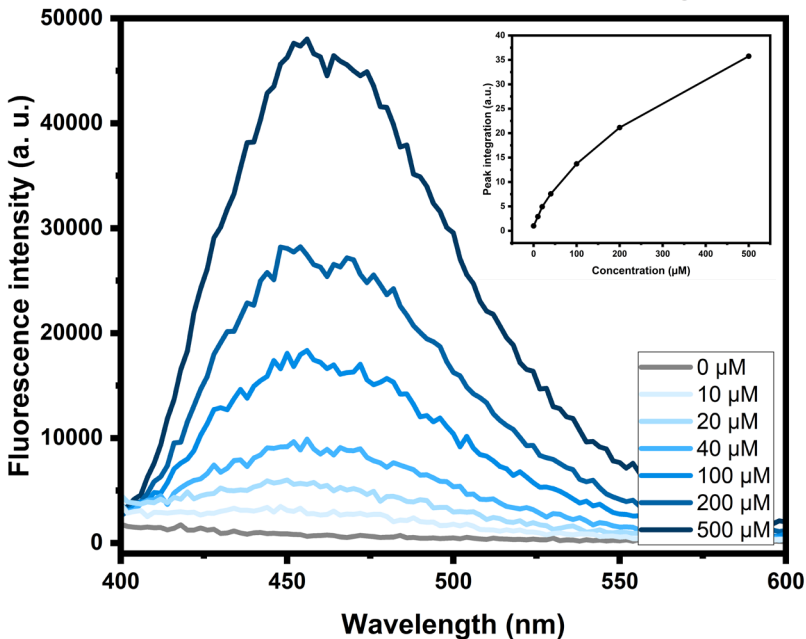

Supplement: Supplemental Fig 14 [file mmc14.pdf]

## Membrane partitioning

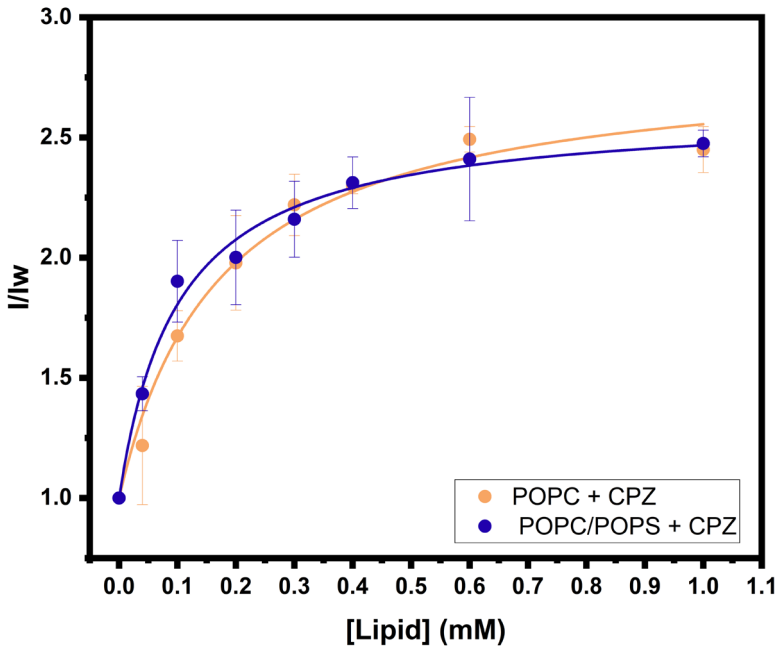

Supplement: Supplemental Fig 15 [file mmc15.pdf]
