# Supplementary material for: The impact of charge on chlorpromazine interaction with lipid membranes
Source: J Lipid Res. 2026 Apr 6;67(5):101035. doi: 10.1016/j.jlr.2026.101035 (PMC13158409; doi:10.1016/j.jlr.2026.101035)
Supplement: Supplemental Fig 11 [file mmc11.pdf]

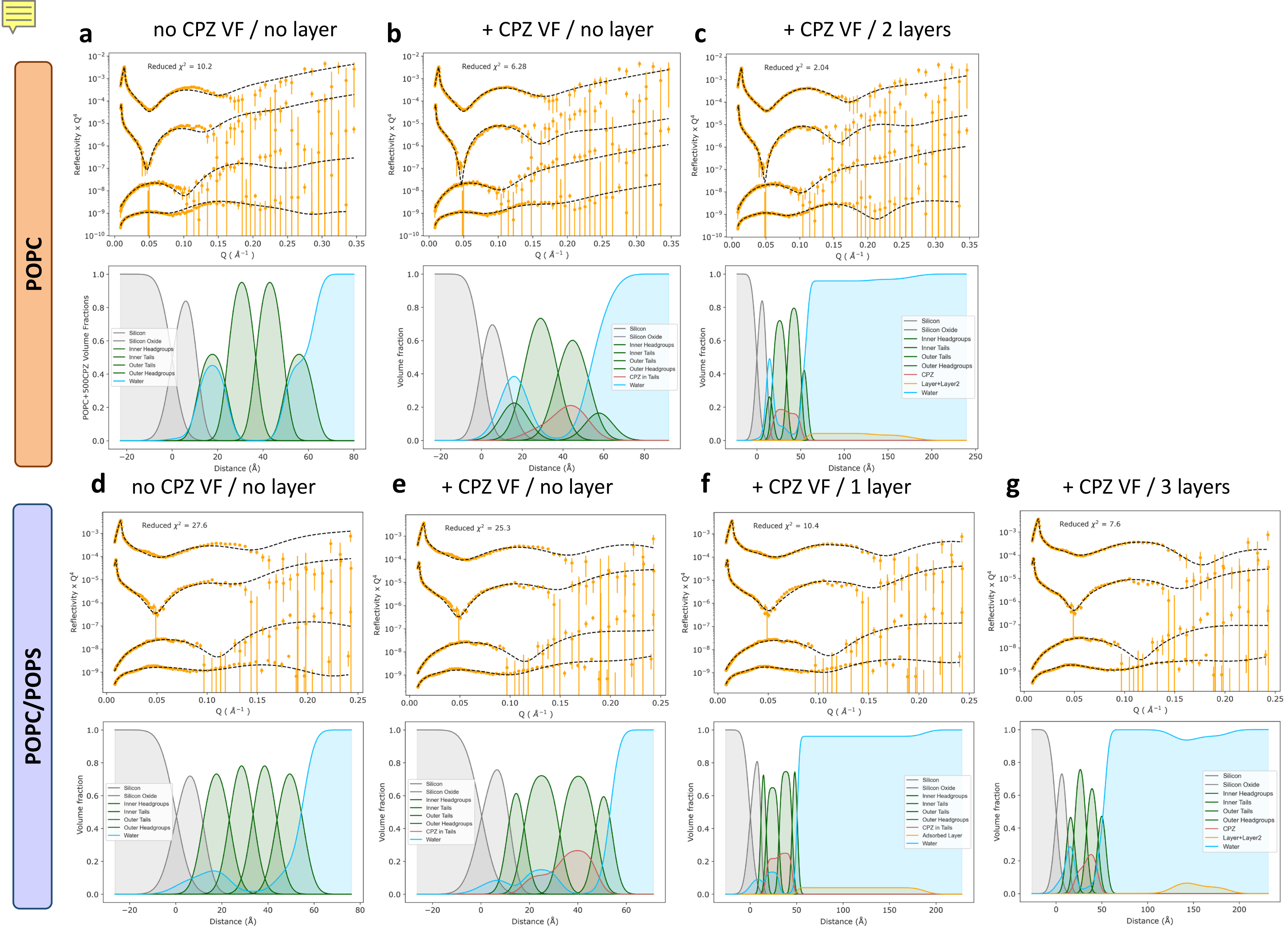

POPC
POPC/POPS
a
no CPZ VF / no layer
b
+ CPZ VF / no layer
c
+ CPZ VF / 2 layers
d
no CPZ VF / no layer
e
+ CPZ VF / no layer
f
+ CPZ VF / 1 layer
g
+ CPZ VF / 3 layers
Reduced χ^2 = 10.2
Reduced χ^2 = 6.28
Reduced χ^2 = 2.04
Reduced χ^2 = 27.6
Reduced χ^2 = 25.3
Reduced χ^2 = 10.4
Reduced χ^2 = 7.6
Reflectivity x Q^4
Q ($Å^{-1}$)
POPC+500CPZ Volume Fractions
Volume fraction
Distance (Å)
Silicon
Silicon Oxide
Inner Headgroups
Inner Tails
Outer Tails
Outer Headgroups
CPZ in Tails
CPZ
Layer+Layer2
Adsorbed Layer
Water
