# Supplementary material for: The impact of charge on chlorpromazine interaction with lipid membranes
Source: J Lipid Res. 2026 Apr 6;67(5):101035. doi: 10.1016/j.jlr.2026.101035 (PMC13158409; doi:10.1016/j.jlr.2026.101035)
Supplement: Supplemental Material [file mmc16.docx]

**SUPPLEMENTAL INFORMATION:**

**The impact of charge on chlorpromazine interaction with lipid membranes**

Ana Gorse^1^, Nicolò Paracini^2,3^, Marion Mathelié-Guinlet^1^, Victoria Lublin^1^, Nada Taib-Maamar^1^, Andrea Valentina Lopez Castillo^1^, Yann Fichou^1^, Estelle Rascol^1*^, Isabel D. Alves^1*^

^1^ Univ. Bordeaux, CNRS, Bordeaux INP, CBMN, UMR 5248, F-33600 Pessac, France

^2^ Institut Laue Langevin, Avenue des Martyrs 71, Grenoble 38000, France

^3^ Current address: Data Management and Software Centre, European Spallation Source ERIC, Asmussens Allé 305, Lyngby 2800, Denmark

**Supplemental Fig. S1: CPZ effect on membrane properties determined using AFM force spectroscopy.** Topographic image of POPC SLB after 1 h incubation with 500 µM CPZ. (a) The histograms representing mechanical stability (b) and rupture depth (c) of POPC bilayer alone before (grey) and after incubation with 500 µM CPZ (orange). The histograms representing POPC/POPS bilayer alone (grey) and after 500 µM CPZ incubation where the dark blue colour represents the first breakthrough event and the light blue colour the second breakthrough event; mechanical stability (f) and rupture depth (g) distributions are displayed. The histograms represent 64 FCs analysed in 3 different areas of the bilayer in order to obtain graphical distribution before and after incubation with 500 µM CPZ. For POPC/POPS bilayer, mechanical stability (d) and rupture depth (e) are shown in the absence (grey) and the presence of 50 µM CPZ where two-step breakthrough event was observed: the first one is displayed in dark blue and the second one in light blue colour. Mean values are displayed, and the error bars represent one-sided standard error of the mean (d, e).

**Supplemental Fig. S2: NR signal stability overtime for POPC lipid bilayer (H_2_O buffer contrast) in the presence of 10 µM CPZ.** The bilayer alone is shown in grey and the bilayer after CPZ injection is shown in orange (light orange: after 1 h, dark orange: after 3 h incubation). The error bars represent the standard deviation in the intensity measured at each q-point, following Poisson counting statistics.

**Supplemental Fig. S3: Summary of the analysis for the POPC bilayer before addition of CPZ.** (a) Neutron reflectivity data measured in four contrasts (points) and model fit lines (dashed black). Data is offset vertically for clarity, from the top, D_2_O, FMW, SMW and H_2_O contrasts. The global reduced χ^2^ value is displayed in the plot. (b) SLD profiles obtained from the fit to the reflectivity data. (c) Corresponding volume fraction profiles of the components in the system and (d) corner plot of the values employed in the model used to fit the reflectivity data, mean values and standard deviations are shown in Supplemental Table S1 and S3. The error bars in the reflectivity data (a) represent the standard deviation in the intensity measured at each q-point, following Poisson counting statistics.

**Supplemental Fig. S4: Summary of the analysis for the POPC bilayer in the presence of 500 µM CPZ fitted with CPZ VF in the tail region and one adsorbed layer.** (a) Neutron reflectivity data measured in four contrasts (points) and model fit lines (dashed black). Data is offset vertically for clarity, from the top, D_2_O, FMW, SMW and H_2_O contrasts. The global reduced χ^2^ value is displayed in the plot. (b) SLD profiles obtained from the fit to the reflectivity data. (c) Corresponding volume fraction profiles of the components in the system and (d) corner plot of the values employed in the model used to fit the reflectivity data, mean values and standard deviations are shown in Supplemental Table S2 and S3. The histogram is a magnified version of the marginalised posterior distribution of the VF ratio of CPZ in the tail region. The volume fractions of CPZ shown here refer to the fraction of the volume occupied by the non-aqueous components of the layer (i.e. 100 % CPZ volume fraction in the tail region would not correspond to the totality of the volume fraction of the layer but to the totality of the volume occupied by the tails, excluding the solvent). The error bars in the reflectivity data (a) represent the standard deviation in the intensity measured at each q-point, following Poisson counting statistics.

**Supplemental Fig. S5: Summary of the analysis for the POPC bilayer in the presence of 500 µM CPZ fitted with CPZ VF in the tail and head group regions and one adsorbed layer.** (a) Neutron reflectivity data measured in four contrasts (points) and model fit lines (dashed black). Data is offset vertically for clarity, from the top, D_2_O, FMW, SMW and H_2_O contrasts. The global reduced χ^2^ value is displayed in the plot. (b) SLD profiles obtained from the fit to the reflectivity data. (c) Corresponding volume fraction profiles of the components in the system and (d) corner plot of the values employed in the model used to fit the reflectivity data, mean values and standard deviations are shown in Supplemental Table S2 and S3. The histogram is a magnified version of the marginalised posterior distribution of the VF ratio of CPZ in the tail and headgroup regions. The volume fractions of CPZ shown here refer to the fraction of the volume occupied by the non-aqueous components of the layer (i.e. 100 % CPZ volume fraction in the tail region would not correspond to the totality of the volume fraction of the layer but to the totality of the volume occupied by the tails, excluding the solvent). The error bars in the reflectivity data (a) represent the standard deviation in the intensity measured at each q-point, following Poisson counting statistics.

**Supplemental Fig. S6: Summary of the analysis for the POPC bilayer after rinsing with buffer fitted with CPZ VF in the tail region and one adsorbed layer.** (a) Neutron reflectivity data measured in four contrasts (points) and model fit lines (dashed black). Data is offset vertically for clarity, from the top, D_2_O, FMW, SMW and H_2_O contrasts. The global reduced χ^2^ value is displayed in the plot. (b) SLD profiles obtained from the fit to the reflectivity data. (c) Corresponding volume fraction profiles of the components in the system and (d) corner plot of the values employed in the model used to fit the reflectivity data, mean values and standard deviations are shown in Supplemental Table S2 and S3. The histogram is a magnified version of the marginalised posterior distribution of the VF ratio of CPZ in the tail region. The volume fractions of CPZ shown here refers to the fraction of the volume occupied by the non-aqueous components of the layer (i.e. 100 % CPZ volume fraction in the tail region would not correspond to the totality of the volume fraction of the layer but to the totality of the volume occupied by the tails, excluding the solvent). The error bars in the reflectivity data (a) represent the standard deviation in the intensity measured at each q-point, following Poisson counting statistics.

**Supplemental Fig. S7: Summary of the analysis for the POPC/POPS bilayer before addition of CPZ.** (a) Neutron reflectivity data measured in four contrasts (points) and model fit lines (dashed black). Data is offset vertically for clarity, from the top, D_2_O, FMW, SMW and H_2_O contrasts. The global reduced χ^2^ value is displayed in the plot. (b) SLD profiles obtained from the fit to the reflectivity data. (c) Corresponding volume fraction profiles of the components in the system and (d) corner plot of the values employed in the model used to fit the reflectivity data, mean values and standard deviations are shown in Supplemental Table S1 and S3. The error bars in the reflectivity data (a) represent the standard deviation in the intensity measured at each q-point, following Poisson counting statistics.

**Supplemental Fig. S8: Summary of the analysis for the POPC/POPS bilayer in the presence of 500 µM CPZ fitted with CPZ VF in the tail region and two adsorbed layers.** (a) Neutron reflectivity data measured in four contrasts (points) and model fit lines (dashed black). Data is offset vertically for clarity, from the top, D_2_O, FMW, SMW and H_2_O contrasts. The global reduced χ^2^ value is displayed in the plot. (b) SLD profiles obtained from the fit to the reflectivity data. (c) corresponding volume fraction profiles of the components in the system and (d) corner plot of the values employed in the model used to fit the reflectivity data, mean values and standard deviations are shown in Supplemental Table S2 and S3. The histogram is a magnified version of the marginalised posterior distribution of the VF ratio of CPZ in the tail region. The volume fractions of CPZ shown here refers to the fraction of the volume occupied by the non-aqueous components of the layer (i.e. 100 % CPZ volume fraction in the tail region would not correspond to the totality of the volume fraction of the layer but to the totality of the volume occupied by the tails, excluding the solvent). The error bars in the reflectivity data (a) represent the standard deviation in the intensity measured at each q-point, following Poisson counting statistics.

**Supplemental Fig. S9: Summary of the analysis for the POPC/POPS bilayer in the presence of 500 µM CPZ fitted with CPZ VF in the tail and head group regions and two adsorbed layers.** (a) Neutron reflectivity data measured in four contrasts (points) and model fit lines (dashed black). Data is offset vertically for clarity, from the top, D_2_O, FMW, SMW and H_2_O contrasts. The global reduced χ^2^ value is displayed in the plot. (b) SLD profiles obtained from the fit to the reflectivity data. (c) Corresponding volume fraction profiles of the components in the system and (d) corner plot of the values employed in the model used to fit the reflectivity data, mean values and standard deviations are shown in Supplemental Table S2 and S3. The histogram is a magnified version of the marginalised posterior distribution of the VF ratio of CPZ in the tail and headgroup regions. The volume fractions of CPZ shown here refers to the fraction of the volume occupied by the non-aqueous components of the layer (i.e. 100 % CPZ volume fraction in the tail region would not correspond to the totality of the volume fraction of the layer but to the totality of the volume occupied by the tails, excluding the solvent). The error bars in the reflectivity data (a) represent the standard deviation in the intensity measured at each q-point, following Poisson counting statistics.

**Supplemental Fig. S10: Summary of the analysis for the POPC/POPS bilayer after rinsing with buffer fitted with CPZ VF in the tail region and one adsorbed layer.** (a) Neutron reflectivity data measured in four contrasts (points) and model fit lines (dashed black). Data is offset vertically for clarity, from the top, D_2_O, FMW, SMW and H_2_O contrasts. The global reduced χ^2^ value is displayed in the plot. (b) SLD profiles obtained from the fit to the reflectivity data. (c) Corresponding volume fraction profiles of the components in the system and (d) corner plot of the values employed in the model used to fit the reflectivity data, mean values and standard deviations are shown in Supplemental Table S2 and S3. The histogram is a magnified version of the marginalised posterior distribution of the VF ratio of CPZ in the tail region. The volume fractions of CPZ shown here refers to the fraction of the volume occupied by the non-aqueous components of the layer (i.e. 100 % CPZ volume fraction in the tail region would not correspond to the totality of the volume fraction of the layer but to the totality of the volume occupied by the tails, excluding the solvent). The error bars in the reflectivity data (a) represent the standard deviation in the intensity measured at each q-point, following Poisson counting statistics.

**Supplemental Fig. S11: Summary of the analysis for the discarded models used to fit the POPC (a-c) and POPC/POPS (d-g) bilayer in the presence of 500 µM CPZ.** The top row shows the reflectivity data measured in four contrasts (points) and model fit lines (dashed black). Data is offset vertically for clarity, from the top, D_2_O, FMW, SMW and H_2_O contrasts. The global reduced χ^2^ value is displayed in the plot. The bottom row shows SLD profiles obtained from the fit to the reflectivity data. The models are: (a, d) simple symmetric bilayers, (b, e) symmetric bilayers including CPZ in the tail region, (c) symmetric POPC bilayer including CPZ in the tail region plus two adsorbed layers above the membrane, (f) symmetric POPC/POPS bilayer including CPZ in the tail region plus one adsorbed layer above the membrane and (d) symmetric POPC/POPS bilayer including CPZ in the tail region plus three adsorbed layers above the membrane. The error bars in the reflectivity data represent the standard deviation in the intensity measured at each q-point, following Poisson counting statistics.

**Supplemental Fig. S12: Representative EPR spectra of TEMPO-PC spin probe in the absence and presence of CPZ.** The first spectra represent POPC-TEMPO vesicles in the absence (black) and the presence of CPZ (blue) with the determined 2A_Z_ parameter (between the maximum of the first and the last peak) (a). The spectra (b) were measured for POPC/POPS-TEMPO vesicles at 20 °C for the vesicles alone (black) and in the presence of CPZ (blue). The ratio of mid-field and high-field spectral lines amplitudes (h_0_/h_−_) is used as a reporter for mobility (the determination of each spectral amplitude is displayed on the graph)*.*

**Supplemental Fig. S13: Effect of CPZ on GP values of POPC (a, orange) and POPC/POPS LUVs (b, blue) determined by Laurdan fluorescence spectroscopy.** The grey dots represent the lipid alone and the blue/orange (from light to dark) represent different CPZ/lipid molar ratios: 1/100, 1/50, 1/25, 1/10 and 1/5.

**Supplemental. Fig. S14: Fluorescence emission intensity of CPZ diluted in buffer at different concentrations and the corresponding integrated peaks on top right.**

**Supplemental Fig. S15: Partition curves of POPC (orange) and POPC/POPS (blue) liposomes in the presence of 40 µM CPZ, obtained by fluorescence spectroscopy measurement.** The fits were obtained using simple partition model, which allowed to obtain the partition coefficient (K_P_). The error bars represent the SD.

**Supplemental Table S1: Fitted values for the lipid bilayers alone.** Parameters obtained from the fits of POPC (left) and POPC/POPS (9/1) (right) lipid bilayers alone that correspond to the analysis shown in Supplemental Fig. S3 and S7). Units of thickness (Thick) and roughness (Rough) are in Å. The volume fractions (VF) are dimensionless and refer to the volume fraction of the components with respect to the whole layer, calculated from the values shown in Supplemental Table S4. The SiO_2_ Hydration parameter corresponds to 1 – VF. The fixed common parameters have the SLD values: SiO_2_ 3.47, headgroup 1.88 and tails 3.17 Å^2^ * 10^-6^. The errors represent 65 % confidence intervals of the marginalised posterior distributions from the Bayesian analysis.

|  | | **POPC** | | | | **POPC/POPS** | | | |
| --- | --- | --- | --- | --- | --- | --- | --- | --- | --- |
| **Bilayer alone** | **Volume Fraction** | **Value** | **Error** | **Lower Bound** | **Upper Bound** | **Value** | **Error** | **Lower Bound** | **Upper Bound** |
|  | **SiO_2_ Thick** | 11.0 | 0.3 | 2 | 30 | 11.8 | 0.5 | 2 | 30 |
|  | **SiO_2_ Rough** | 4.5 | 0.4 | 0 | 7 | 5.3 | 0.3 | 0 | 7 |
|  | **SiO_2_ Hydration (1-VF)** | 0.02 | 0.02 | 0 | 0.5 | 0.099 | 0.02 | 0 | 0.5 |
|  | **Water Layer Thick** | 2.6 | 0.4 | 0 | 30 | - | - | - | - |
|  | **Head Group Thick** | 9.4 | 0.6 | 0 | 15 | 7.2 | 0.3 | 0 | 15 |
|  | **Head Group VF** | 0.45 | 0.02 | 0.3 | 1 | 0.56 | 0.02 | 0.3 | 1 |
|  | **Tails Thick** | 17.1 | 0.2 | 10 | 20 | 18.3 | 0.2 | 10 | 20 |
|  | **Tails VF** | 0.99 | 0.01 | 0.5 | 1 | 0.91 | 0.01 | 0.5 | 1 |
|  | **Bilayer Rough** | 2.5 | 0.3 | 2 | 10 | 2.6 | 0.4 | 2 | 10 |

**Supplemental Table S2: Fitted values for the lipid bilayers after incubation with 500 µM CPZ and rinsing, where CPZ is fitted in the tail region / tail and headgroup region.** Parameters obtained from the fits of POPC (left) and POPC/POPS (9/1) (right) lipid bilayers correspond to the analysis shown in Supplemental Fig. S4-S6 and S8-S10). Units of thickness (Thick) and roughness (Rough) are in Å. The volume fractions (VF) are dimensionless and refer to the volume fraction of the components with respect to the whole layer, calculated from the values shown in Supplemental Table S4. The fixed common parameters have the SLD values: SiO_2_ 3.47, headgroup 1.88, tails 3.17 and CPZ 1.30 Å^2^ * 10^-6^. The errors associated with the VF are displayed in the Supplemental Table S4. The errors represent 65 % confidence intervals of the marginalised posterior distributions from the Bayesian analysis.

|  | | **POPC** | | | | **POPC/POPS** | | | | |
| --- | --- | --- | --- | --- | --- | --- | --- | --- | --- | --- |
|  | **Volume Fraction** | **Value** | **Error** | **Lower Bound** | **Upper Bound** | **Value** | **Error** | **Lower Bound** | **Upper Bound** |  |
| **+ 500 µM CPZ (CPZ in tails)** | **Head Group Thick** | 7.1 | 0.5 | 5 | 15 | 8.4 | 0.8 | 5 | 15 |  |
|  | **Tails Thick** | 16.0 | 0.3 | 10 | 20 | 12.6 | 0.8 | 10 | 20 |  |
|  | **Bilayer Rough** | 4.2 | 0.5 | 2 | 10 | 2.4 | 0.4 | 2 | 10 |  |
|  | **Ads. Layer Thick** | 119.0 | 2.0 | 0 | 150 | 65.9 | 0.9 | 0 | 100 |  |
|  | **Ads. Layer SLD** | 2.31 | 0.08 | 1.3 | 4 | 2.96 | 0.75 | 1.3 | 4 |  |
|  | **Ads. Layer Rough** | 14.7 | 0.4 | 3 | 15 | 7.6 | 1.7 | 3 | 15 |  |
|  | **Ads. Layer VF** | 0.04 | 0.01 | 0 | 0.2 | 0.00 | 0.00 | 0 | 0.2 |  |
|  | **Ads. Layer 2 Thick** | - | - | - | - | 61.0 | 1.2 | 0 | 100 |  |
|  | **Ads. Layer 2 SLD** | - | - | - | - | 2.26 | 0.03 | 1.3 | 4 |  |
|  | **Ads. Layer 2 Rough** | - | - | - | - | 14.0 | 0.83 | 3 | 15 |  |
|  | **Ads. Layer 2 VF** | - | - | - | - | 0.06 | 0.00 | 0 | 0.2 |  |
| **After rinse (CPZ in tails)** | **Head Group Thick** | 11.8 | 0.3 | 5 | 15 | 5.5 | 0.4 | 5 | 15 |  |
|  | **Tails Thick** | 15.5 | 0.3 | 10 | 20 | 18.6 | 0.5 | 10 | 20 |  |
|  | **Bilayer Rough** | 3.6 | 0.5 | 2 | 10 | 4.0 | 0.7 | 2 | 10 |  |
|  | **Ads. Layer Thick** | 124.0 | 2.9 | 0 | 150 | 19.4 | 7.6 | 0 | 150 |  |
|  | **Ads. Layer SLD** | 1.58 | 0.22 | 1.3 | 3.5 | 2.06 | 0.66 | 1.3 | 4 |  |
|  | **Ads. Layer Rough** | 13.2 | 1.8 | 3 | 15 | 9.5 | 3.9 | 3 | 15 |  |
|  | **Ads. Layer VF** | 0.02 | 0.001 | 0 | 0.2 | 0.03 | 0.02 | 0 | 0.2 |  |
| **+ 500 µM CPZ (CPZ in tails and heads)** | **Head Group Thick** | 6.8 | 0.5 | 5 | 15 | 7.3 | 0.5 | 5 | 15 |  |
|  | **Tails Thick** | 16.5 | 0.4 | 10 | 20 | 13.5 | 0.5 | 10 | 20 |  |
|  | **Bilayer Rough** | 4.3 | 0.3 | 2 | 10 | 2.5 | 0.4 | 2 | 10 |  |
|  | **Ads. Layer Thick** | 120.0 | 2.0 | 0 | 150 | 68.1 | 1.6 | 0 | 100 |  |
|  | **Ads. Layer SLD** | 2.29 | 0.08 | 1.3 | 4 | 3.04 | 0.69 | 1.3 | 4 |  |
|  | **Ads. Layer Rough** | 14.7 | 0.4 | 3 | 15 | 3.7 | 0.6 | 3 | 15 |  |
|  | **Ads. Layer VF** | 0.04 | 0.01 | 0 | 0.2 | 0.00 | 0.00 | 0 | 0.2 |  |
|  | **Ads. Layer 2 Thick** | - | - | - | - | 57.7 | 2.3 | 0 | 100 |  |
|  | **Ads. Layer 2 SLD** | - | - | - | - | 2.26 | 0.02 | 1.3 | 4 |  |
|  | **Ads. Layer 2 Rough** | - | - | - | - | 13.3 | 1.3 | 3 | 15 |  |
|  | **Ads. Layer 2 VF** | - | - | - | - | 0.06 | 0.01 | 0 | 0.2 |  |

**Supplemental Table S3: Bilayer composition after incubation with 500 µM CPZ and rinsing, where CPZ is fitted in the tail region / tail and headgroup region.** The bilayer % compositions are dimensionless and refer to the volume fraction of the components with respect to the whole layer, calculated from the values shown in Supplemental Table S4.

|  | | **POPC** | | | **POPC/POPS** | | |
| --- | --- | --- | --- | --- | --- | --- | --- |
|  | **Bilayer composition** | **% Lipid** | **% CPZ** | **% Water** | **% Lipid** | **%**  **CPZ** | **% Water** |
| **+ 500 µM CPZ (CPZ in tails)** | **Inner Head Group** | 32.0 | NA | 68.0 | 64.0 | NA | 36.0 |
|  | **Inner Tails** | 71.9 | 19.1 | 9.0 | 83.3 | 14.7 | 2.0 |
|  | **Outer Tails** | 82.3 | 15.7 | 2.0 | 69.0 | 23.0 | 8.0 |
|  | **Outer Head Group** | 61.0 | NA | 39.0 | 66.0 | NA | 34.0 |
| **After rinse (CPZ in tails)** | **Inner Head Group** | 37.0 | NA | 63.0 | 57.0 | NA | 43.0 |
|  | **Inner Tails** | 97.0 | 1.0 | 2.0 | 73.1 | 11.9 | 15.0 |
|  | **Outer Tails** | 97.0 | 1.0 | 2.0 | 92.2 | 4.8 | 3.0 |
|  | **Outer Head Group** | 43.0 | NA | 57.0 | 68.0 | NA | 32.0 |
| **+ 500 µM CPZ (CPZ in tails and heads)** | **Inner Head Group** | 27.5 | 4.5 | 68.0 | 51.6 | 13.4 | 35.0 |
|  | **Inner Tails** | 71.1 | 18.9 | 10.0 | 78.0 | 16.0 | 6.0 |
|  | **Outer Tails** | 82.2 | 16.8 | 1.0 | 73.9 | 22.1 | 4.0 |
|  | **Outer Head Group** | 42.9 | 10.1 | 47.0 | 41.6 | 24.4 | 34.0 |

**Supplemental Table S4: Errors associated with the VF of lipids and CPZ.** VF of bilayers and CPZ components obtained from the fits. In the model, the VF of CPZ was calculated as a fraction of the dry component of each layer (excluding the water) for convenience during the fitting procedure. Values shown in Supplemental Tables S1-S3 are calculated from these values, which are the output of the fits. The errors represent 65 % confidence intervals of the marginalised posterior distributions from the Bayesian analysis.

|  | | **POPC** | | | | **POPC/POPS** | | | |
| --- | --- | --- | --- | --- | --- | --- | --- | --- | --- |
|  | **Volume Fraction** | **Value** | **Error** | **Lower Bound** | **Upper Bound** | **Value** | **Error** | **Lower Bound** | **Upper Bound** |
| **+ 500 µM CPZ (CPZ in tails)** | **Inner Head Group** | 0.32 | 0.02 | 0.3 | 0.7 | 0.64 | 0.02 | 0.3 | 0.7 |
|  | **Outer Head Group** | 0.61 | 0.07 | 0.3 | 0.7 | 0.66 | 0.04 | 0.3 | 0.7 |
|  | **Total Inner Tails + CPZ** | 0.91 | 0.02 | 0.5 | 1 | 0.98 | 0.02 | 0.5 | 1 |
|  | **CPZ inner tails** | 0.21 | 0.01 | 0 | 0.7 | 0.15 | 0.03 | 0 | 0.7 |
|  | **Total Outer Tails + CPZ** | 0.98 | 0.02 | 0.5 | 1 | 0.92 | 0.02 | 0.5 | 1 |
|  | **CPZ outer tails** | 0.16 | 0.02 | 0 | 0.7 | 0.25 | 0.02 | 0 | 0.7 |
| **After rinse (CPZ in tails)** | **Inner Head Group** | 0.37 | 0.01 | 0.3 | 0.7 | 0.57 | 0.04 | 0 | 1 |
|  | **Outer Head Group** | 0.43 | 0.02 | 0.3 | 0.7 | 0.68 | 0.12 | 0 | 1 |
|  | **Total Inner Tails + CPZ** | 0.97 | 0.02 | 0.5 | 1 | 0.85 | 0.02 | 0.5 | 1 |
|  | **CPZ inner tails** | 0.01 | 0.01 | 0 | 0.7 | 0.14 | 0.02 | 0 | 0.7 |
|  | **Total Outer Tails + CPZ** | 0.97 | 0.02 | 0.5 | 1 | 0.97 | 0.02 | 0.5 | 1 |
|  | **CPZ outer tails** | 0.01 | 0.01 | 0 | 0.7 | 0.05 | 0.02 | 0 | 0.7 |
| **+ 500 µM CPZ (CPZ in tails and heads)** | **Total Inner Head Group + CPZ** | 0.32 | 0.02 | 0.3 | 0.7 | 0.64 | 0.03 | 0.3 | 0.7 |
|  | **CPZ inner head group** | 0.14 | 0.14 | 0 | 0.5 | 0.21 | 0.13 | 0 | 0.5 |
|  | **Total Inner Tails + CPZ** | 0.90 | 0.02 | 0.5 | 1 | 0.94 | 0.02 | 0.5 | 1 |
|  | **CPZ inner tails** | 0.21 | 0.02 | 0 | 0.7 | 0.17 | 0.03 | 0 | 0.7 |
|  | **Total Outer Tails + CPZ** | 0.99 | 0.02 | 0.5 | 1 | 0.96 | 0.02 | 0.5 | 1 |
|  | **CPZ outer tails** | 0.17 | 0.02 | 0 | 0.7 | 0.23 | 0.02 | 0 | 0.7 |
|  | **Total Outer Head Group + CPZ** | 0.53 | 0.10 | 0.3 | 0.7 | 0.66 | 0.04 | 0.3 | 0.7 |
|  | **CPZ outer head group** | 0.19 | 0.15 | 0 | 0.5 | 0.37 | 0.10 | 0 | 0.5 |

**Supplemental Table S5. Average area per lipid and membrane thickness during 500 ns MD simulations.** The table represents data of the lipid systems POPC and POPC/POPS simulated alone (boxes with 25, 50 and 100 phospholipids measured) and in the presence of 1 CPZ molecule.

|  | **AP presence** | **Lipid molecule number** | **Av. lipid area with SD (Å^2^)** | **Av. membrane thickness with SD (Å)** |
| --- | --- | --- | --- | --- |
| **POPC** | - CPZ | 25 | 63.2 ± 9.8 | 39.4 ± 1.3 |
|  |  | 50 | 63.7 ± 10.5 | 39.2 ± 1.0 |
|  |  | 100 | 63.6 ± 10.8 | 39.2 ± 0.8 |
|  | + CPZ | 25 | 64.4 ± 10.7 | 39.1 ± 1.4 |
|  |  | 50 | 64.1 ± 10.9 | 39.0 ± 1.1 |
|  |  | 100 | 64.0 ± 11.1 | 39.1 ± 0.7 |
| **POPC/POPS** | - CPZ | 25 | 62.0 ± 9.7 | 40.0 ± 1.2 |
|  |  | 50 | 62.9 ± 10.4 | 39.4 ± 0.9 |
|  |  | 100 | 62.9 ± 10.8 | 39.4 ± 0.6 |
|  | + CPZ | 25 | 64.4 ± 10.7 | 39.1 ± 1.4 |
|  |  | 50 | 64.1 ± 10.9 | 37.7 ± 1.0 |
|  |  | 100 | 63.4 ± 10.9 | 39.2 ± 0.8 |

**Supplemental Table S6. Partition coefficient (K_P_) of CPZ (0.04 mM) obtained using fluorescence spectroscopy by increasing POPC or POPC/POPS liposome concentration.** The experiments were done at room T, and the K_P_ for POPC/POPS liposomes is significantly higher than for POPC liposomes (Welch’s T-test, p=0.03).

| **Lipid system** | **Partition coefficient with SD (* 10^4^)** |
| --- | --- |
| **POPC** | 0.75 ± 0.14 |
| **POPC/POPS** | 1.28 ± 0.22 |
