# Supplementary material for: The impact of charge on chlorpromazine interaction with lipid membranes
Source: J Lipid Res. 2026 Apr 6;67(5):101035. doi: 10.1016/j.jlr.2026.101035 (PMC13158409; doi:10.1016/j.jlr.2026.101035)
Supplement: Supplemental Fig 6 [file mmc6.pdf]

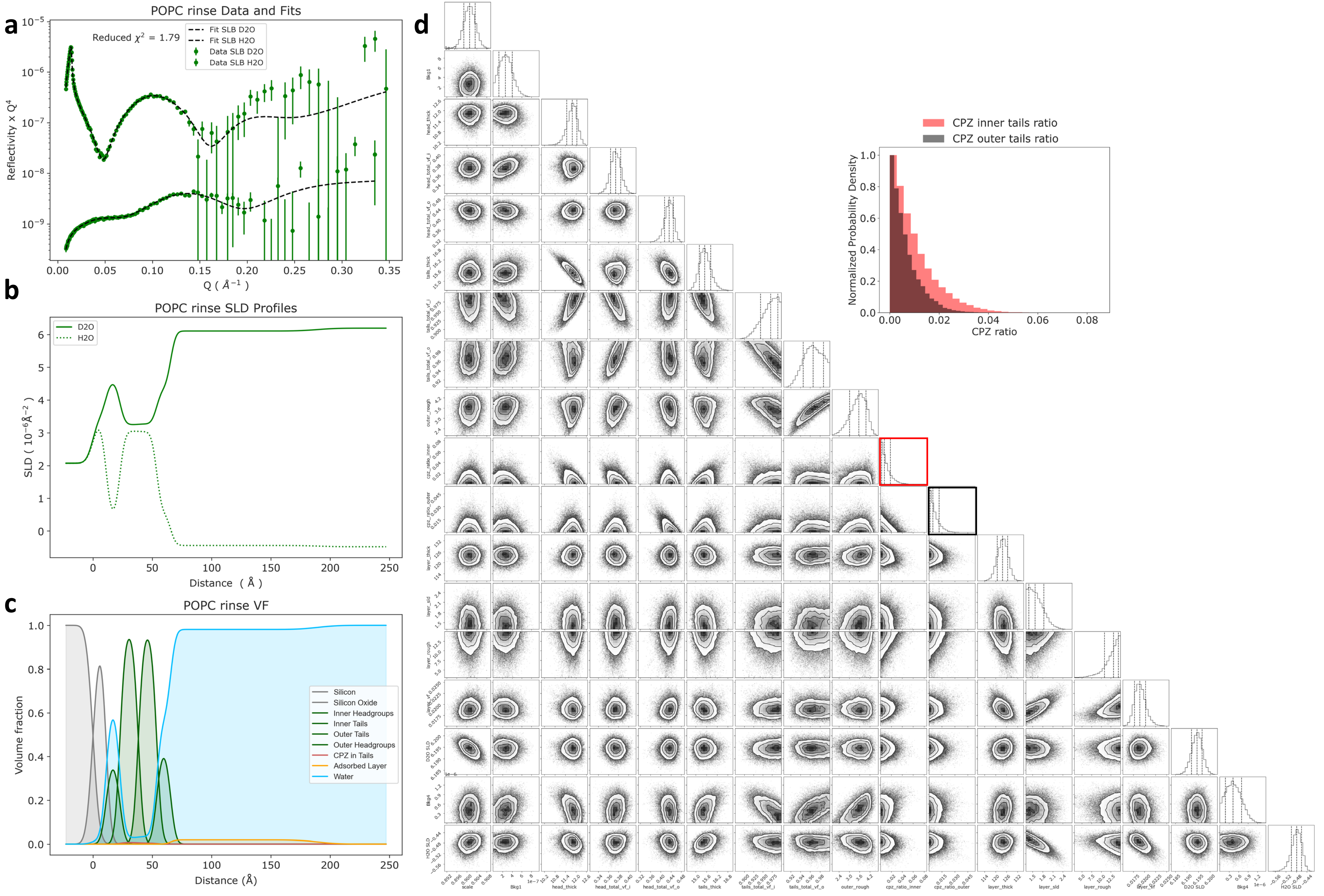

a
POPC rinse Data and Fits
Reduced χ^2 = 1.79
Fit SLB D2O
Fit SLB H2O
Data SLB D2O
Data SLB H2O
Reflectivity x Q^4
Q (Å^{-1})
b
POPC rinse SLD Profiles
D2O
H2O
SLD (10^{-6}Å^{-2})
Distance (Å)
c
POPC rinse VF
Silicon
Silicon Oxide
Inner Headgroups
Inner Tails
Outer Tails
Outer Headgroups
CPZ in Tails
Adsorbed Layer
Water
Volume fraction
Distance (Å)
d
CPZ inner tails ratio
CPZ outer tails ratio
Normalized Probability Density
CPZ ratio
scale
Bkg1
head_thick
head_total_vf_i
head_total_vf_o
tails_thick
tails_total_vf_i
tails_total_vf_o
outer_rough
cpz_ratio_inner
cpz_ratio_outer
layer_thick
layer_sld
layer_rough
layer_vf
D2O SLD
Bkg4
H2O SLD
